# Bayesian Estimation of Individual Gray Whale Space Use Reveals Differential Exposure to Stressors

**DOI:** 10.1002/ece3.71330

**Published:** 2025-05-25

**Authors:** Lisa Hildebrand, Leslie New, Enrico Pirotta, Joshua D. Stewart, Ines Hildebrand, Carrie Newell, K. C. Bierlich, Clara N. Bird, Alejandro Fernandez Ajó, Daniel Turek, Leigh G. Torres

**Affiliations:** ^1^ Geospatial Ecology of Marine Megafauna Lab, Marine Mammal Institute, Department of Fisheries, Wildlife and Conservation Sciences Oregon State University Newport Oregon USA; ^2^ Department of Mathematics, Computer Science and Statistics Ursinus College Collegeville Pennsylvania USA; ^3^ Centre for Research into Ecological and Environmental Modelling University of St Andrews St Andrews Scotland UK; ^4^ Ocean Ecology Lab, Marine Mammal Institute, Department of Fisheries, Wildlife and Conservation Sciences Oregon State University Newport Oregon USA; ^5^ Whale Research EcoExcursions Depoe Bay Oregon USA; ^6^ Department of Mathematics Lafayette College Easton Pennsylvania USA

**Keywords:** baleen whales, Bayesian modeling, conservation, individual variation, Pacific coast feeding group, space use, spatially explicit capture‐recapture, stressor exposure

## Abstract

This study quantifies the individual space use patterns of Pacific Coast Feeding Group gray whales ( *Eschrichtius robustus* ) from photographic capture‐recapture data, collected in central Oregon, U.S.A., within a Bayesian framework. We evaluate the potential exposure of individuals to six anthropogenic stressors given their space use patterns. We used an 8‐year dataset of spatially explicit encounter histories collected via photo‐identification during continuous boat surveys to inform a Bayesian spatially explicit capture‐recapture model and estimate space use of individual whales. Space use estimates were combined with exposure values of four static (distance from two ports, distance from an effluent discharge site, area of whale watching) and two dynamic (commercial Dungeness crab pots, recreational fishing) anthropogenic stressors or their proxies to estimate relative individual stressor exposure. The influence of age and sex on space use patterns and stressor exposure was assessed *post hoc*. Space use, and thereby stressor exposure, was highly variable among individuals, both within and between years. Some individuals displayed remarkable long‐term and fine‐spatial‐scale site fidelity, not typically documented for large baleen whales. Juveniles concentrate their space use in a distinct area that is proximal to a port and center of whale watch activity. Exposure to stressors is highly variable across individuals and years given the heterogeneity of individual space use within the population and of stressor distribution, underscoring the complexity of managing wildlife populations. While population management plans need to be implemented at a population level, the recognition and incorporation of intraspecific variation can improve regulation efficacy since individual performance has relevant consequences on population health.

## Graphical abstract

Pacific Coast Feeding Group gray whale individuals show high variability in space use within and between years, which results in differences in exposure to six anthropogenic stressors. Some individuals show remarkably high and long‐term site fidelity to small spatial areas (< 20 km in length). We were unable to identify one leading stressor that all individuals were exposed to, revealing the complexity of managing wildlife populations.

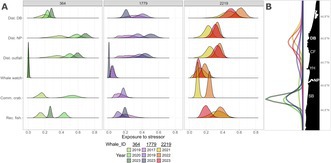

## 1 Introduction

Negative impacts of human activities on wildlife populations are extensively documented across ecosystems and taxa (Coetzee and Chown 2016; Scanes 2018; Hill et al. 2019; Kok et al. 2023). Effective management and conservation require knowledge of a population's exposure to stressors across space and time (Burger and Shaffer 2008; Brooks et al. 2019). Spatially explicit assessments of exposure have been widely implemented, yet most are conducted at a population level. However, studies across taxa show that individuals within a population often have heterogenous space use patterns (Schofield et al. 2010; Gutowsky et al. 2015; Allen et al. 2016; De Moura et al. 2021). Individual space use can vary due to differences in sex (Bixler and Gittleman 2000), age, reproductive stage (van Beest et al. 2011), personality (Schirmer et al. 2019), resource availability (Svoboda et al. 2019), and risk perception (Lima and Dill 1990). Thus, creating management plans based on population‐level space use could impact their efficacy for different segments of the population (Pirotta et al. 2018), such as individuals in vulnerable life‐history stages (e.g., juveniles, pregnant or lactating females) that may be at higher risk of exposure to stressors (Griffin et al. 2017; Keen et al. 2021). Frameworks like the population consequences of multiple stressors (PCoMS) provide a pathway to integrate individual‐level data on physiological, behavioral and health responses to stressors that may affect an individual's vital rates into an assessment of population dynamics (National Academies 2017; Tyack et al. 2022). Yet, to estimate an individual's response to a stressor, we must first understand how its exposure rates vary in space and time, which relies on accurate assessment of individual distribution and habitat use patterns.

Marine environments are increasingly exploited as global demands for food security, renewable energy, commercial shipping, and recreation continue to rise, all of which threaten marine wildlife (McKay and Mulvaney 2001; Crain et al. 2009). These anthropogenic activities are not distributed homogenously in space (Halpern et al. 2008). Thus, understanding how populations are distributed relative to anthropogenic use of the marine environment is critical for management efforts. Species distribution models are a common tool used to map wildlife distribution (Austin 2007; Elith and Leathwick 2009), which enable co‐occurrence analysis with spatial data on anthropogenic stressors to determine overlap rates across space or time. These methods have successfully mapped the risk of multiple anthropogenic stressors with a wide variety of marine species (e.g., Torres et al. 2013; Krüger et al. 2018; Zhang et al. 2024). Data to build species distribution models typically are collected using survey methods that do not lend themselves to individual identification. Therefore, these modeling approaches estimate distribution and exposure at the population (rather than individual) level, which could fail to identify individuals that are experiencing extreme levels of stressor exposure.

Individual space use patterns can be analyzed using spatially explicit capture‐recapture (SECR) methods, where individual capture histories are combined with spatial information on capture locations (Borchers and Efford 2008; Royle et al. 2009a, 2009b). These methods were initially developed for gridded camera‐trap studies in terrestrial systems (e.g., Royle et al. 2009a; Green et al. 2020) where the probability of capturing an individual at any given trap varies given its distribution with respect to the trap. Individuals are assumed to have an activity center where the probability of capture is highest. The likelihood of capturing an individual declines with increasing distance from its center following a specified detection function (Efford 2004). Thus, SECR methods enable the estimation of encounter probabilities for individuals in a population across a study area, which can be interpreted as an individual's space use (e.g., Allen et al. 2016; Cayuela et al. 2017) that can then be compared to the distribution of spatiotemporally aligned stressors to determine exposure. This approach has been successfully adapted to continuous survey data to quantify variability in individual stressor exposure in marine populations (Christiansen et al. 2015; Pirotta et al. 2015; Glennie et al. 2021).

The Pacific Coast Feeding Group (PCFG) is a gray whale ( *Eschrichtius robustus* ; ~212 individuals, Harris et al. 2022) subgroup that migrates from wintering grounds in Baja California Peninsula, Mexico, to feeding grounds along the Pacific Northwest coast of North America (41° N‐52° N). Within their feeding range, PCFG whales display a strong affinity for nearshore, shallow (< 20 m) habitats and high fidelity to specific areas within the range intra/interannually (Calambokidis et al. 2002; Barlow et al. 2024). Satellite tagging of PCFG whales reveals long residence times (32–65 days) in areas along the Oregon coast, USA (Lagerquist et al. 2019). Several anthropogenic activities occur in this foraging region that may impact PCFG whales. Males show increased glucocorticoid concentrations associated with increased sound levels the previous day, which are correlated with increased vessel traffic (Lemos et al. 2022; Pirotta et al. 2023). Individuals in close proximity (< 250 m) to vessels are likely to transition from search to travel behavior, possibly compromising feeding opportunities (Sullivan and Torres 2018). Entanglements in pot fisheries and vessel strikes result in lethal and nonlethal injuries (Scordino et al. 2020; Walsh et al. 2024). Furthermore, the age structure of the PCFG indicates the potential for an impending population decline (low numbers of juveniles relative to adults; Pirotta et al. in press). While these studies demonstrate collective exposure of the PCFG to multiple stressors, knowledge of individual‐level stressor exposure does not exist. Our goal was to describe space use and stressor exposure while individuals are in our study area, rather than the drivers of their occurrence along this coast. Therefore, for individual whales observed frequently in our study area during surveys conducted over 8 years (2016–2023), we (1) use a Bayesian SECR model to estimate annual space use and subsequently (2) evaluate the potential exposure of individuals to six anthropogenic stressors, given their space use patterns. Our application of a SECR model to continuous survey data demonstrates an effective approach for considering individual space use and stressor exposure within the context of population‐level wildlife management.

## 2 Methods

### 2.1 Data Collection

**FIGURE 1:**
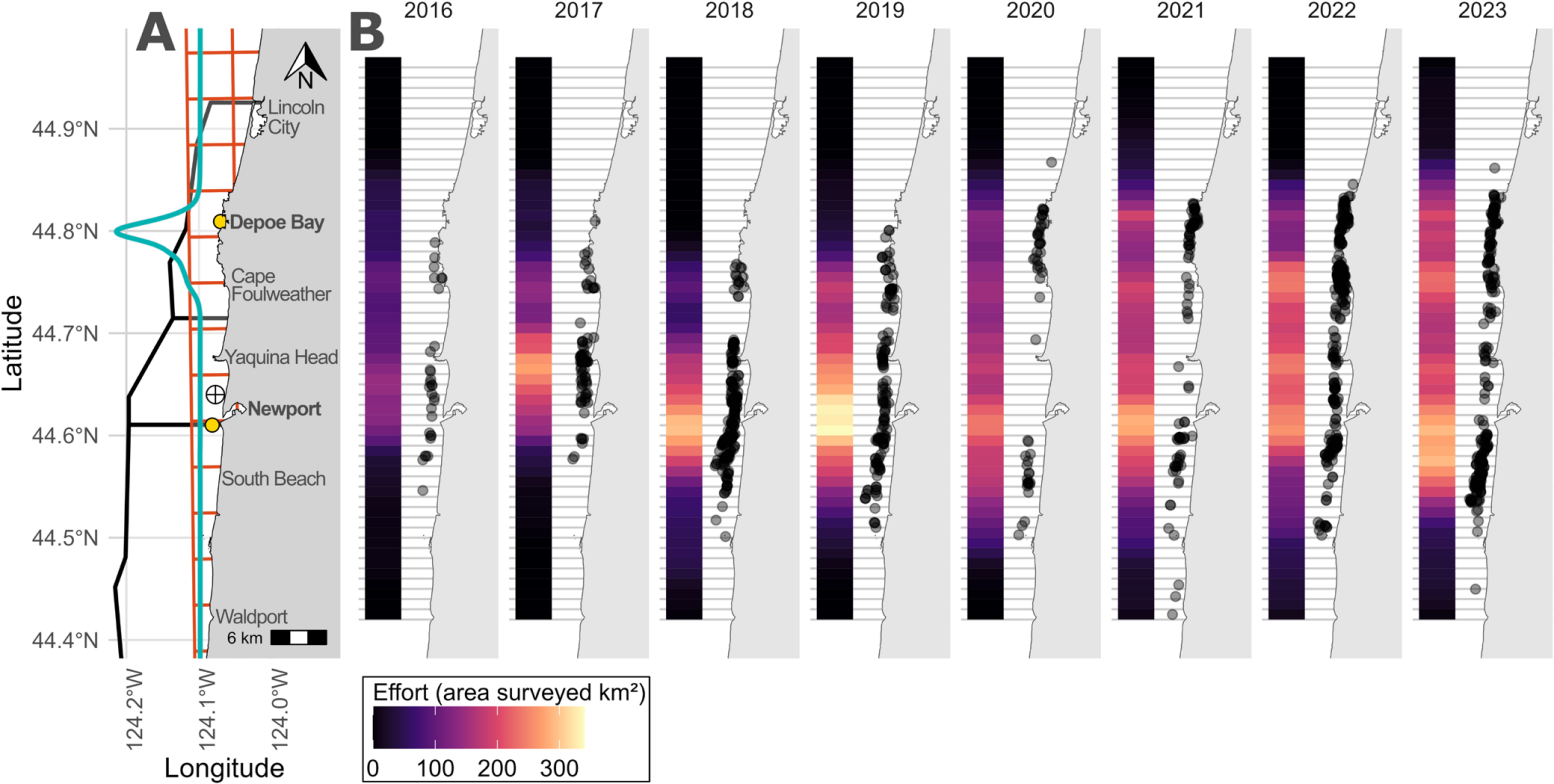
(A) Map of the study area depicting different spatial landmarks and anthropogenic stressors. The black lines demarcate the three recreational fishing zones, the orange grid demarcates the 5 × 5 km commercial crab pot grid cells, the teal distribution demarcates distribution of whale watching activity, the yellow circles indicate the locations of Depoe Bay and Newport ports, and the cross symbol indicates the site where the effluent discharge line ends. (B) Spatial encounters (black circles) of individuals included in the spatially explicit capture‐recapture model for each year of the study period (2016–2023). Circles are plotted with transparency to visualize density of encounters, with overlapping circles appearing darker (black) and less dense circles appearing lighter (gray). Latitude bins are depicted by light gray horizontal lines. The amount of area surveyed (km^2^) each year within each latitude bin is represented via a color ramp from no effort (black) to highest effort (yellow).

Data were collected under NMFS permits #16111 and #21678 issued to John Calambokidis, and #27246 issued to the Marine Mammal Institute at Oregon State University. Authorization for this noninvasive research was provided by the Oregon State University Institutional Animal Care and Use Committee. Photographs and spatial encounters of gray whales were collected through systematic surveys along the central Oregon coast, United States (60 km; Figure 1) from a rigid‐hulled inflatable boat (5.4 m) when weather allowed from June to mid‐October 2016–2023. Upon sighting whales, animals were approached to obtain photographs for identification and a precise location. Unique pigmentation and scarring of gray whales allow for noninvasive photographic resightings of individuals through time. Data collection was part of a larger project (see Lemos et al. 2022). Observation conditions (Beaufort Sea State, swell, visibility) were continuously recorded during survey effort and used to calculate spatial survey effort (km^2^; Text S1, Table S1) for each survey day within each latitude bin.

### 2.2 Spatially Explicit Capture‐Recapture Model

We used a Bayesian SECR approach to estimate annual activity centers and ranges for each individual in our study area. The temporal scale of the model was at the annual level because sample sizes limited space use estimation at a finer temporal scale. Data collected via photo‐identification surveys can be adapted to the SECR framework by gridding the sampling area and treating each grid cell as a sampling unit (e.g., Christiansen et al. 2015; Pirotta et al. 2015; Glennie et al. 2021). Given the very nearshore distribution of PCFG whales, with little longitudinal variation in their sighting distribution (Figure 1), we divided our study area into a one‐dimensional (latitude only) grid comprising 55 latitudinal bins, each spanning 0.01° of latitude (1.1 km in distance) and assigned each spatial encounter to a bin. The number of encounters per latitude bin per year was used to build spatially explicit encounter histories for each individual. We only included encounter histories for individuals seen ≥ five survey days in a year, to provide sufficient information for model estimation of space use parameters. Individuals seen < five survey days in a year were excluded from the model. Sightings were assumed to be Poisson distributed, *S*_*i,l,t*_ ~ Poisson($\lambda_{i,l,t}$), where *S*_*i,l,t*_ indicates how many times individual *i* was sighted in latitude bin *l* in year *t*. $\lambda_{i,l,t}$ is the expected sighting rate for *i* in *l* in *t*, which decays following a half‐normal function of the distance between the centroid of each latitude bin and the annual activity center (*C*_*i,t*_) of individual *i* in year *t*, $d_{i,l,t}$, to yield

$$\lambda_{i,l,t}=\left\{\begin{array}{cc} 0 & \text{if effort}=0\\ \gamma_{i,l,t}\times \exp\left(-d_{i,l,t}^{2}/{2\sigma }_{i,t}^{2}\right) & \text{otherwise} \end{array}\right\}$$

where $\gamma_{i,l,t}$ is the baseline sighting rate for *i* in *l* in *t*, given effort (see below), and $\sigma_{i,t}^{2}$ defines the activity range for *i* in *t*, thus determining the rate at which detection probability declines with distance from the activity center. The formulation of $\lambda_{i,l,t}$ is a step function such that when effort is 0, then the expected sighting rate of an individual is 0 as well. An individual's annual activity center (*C*_*i,t*_) is an estimated variable representing the latitude at which an individual's activity concentrates. This variable had a uniform prior distribution, which constrained an individual's one‐dimensional center of activity to occur in one of the 55 latitude bins within the study area. $\gamma_{i,l,t}$ itself is a function of effort (scaled by dividing by the maximum effort and multiplying by 10) and a random effect for individual and year:

$$\gamma_{i,l,t}=A_{i,t}\times \exp\left(R_{i,t}+\beta_{e}\times e_{l,t}\right)$$

where $e_{l,t}$ represents the area surveyed (km^2^) in *l* and *t*, $\beta_{e}$ is the associated slope parameter, $R_{i,t}$ represents the random effect for individual and year with standard deviation 1, and $A_{i,t}$ is a binary term indicating whether an individual was estimated to be in the study area in that year, with an uninformative prior assuming a 0.5 probability of presence, $A_{i,t}$ ~ *Bernoulli* (0.5). If an individual was estimated to be outside the study area, then their activity center was arbitrarily fixed at 52° N and activity range at 0.001. This ensured that the high level of uncertainty associated with unseen individuals did not affect space use parameter estimation for individuals observed within the study area. The activity range ($\sigma_{i,t}^{2}$) is determined as follows

$$\log\left(\sigma_{i,t}^{2}\right)=\mu +M_{i,t}$$

where μ is a population mean activity range and $M_{i,t}$ represents the random effect for individual and year with standard deviation ω.

The model was fitted using Markov chain Monte Carlo (MCMC) algorithms implemented in package NIMBLE v1.1.0 (de Valpine et al. 2017; NIMBLE Development Team 2024) in R v4.3.2 (R Core Team 2024; Table S2 for model parameter prior distributions, and Text S2 and Figure S1 for a prior sensitivity analysis). Three chains were iterated for 400,000 iterations with a burn‐in of 50,000 iterations. Chains were thinned to manageable object sizes by retaining 1 in 100 iterations. Convergence and mixing were assessed visually by inspecting trace and density plots. Additionally, we ensured the Brooks‐Gelman‐Rubin diagnostic fell below 1.1 and the effective sample size was > 400 for all parameters (Lunn et al. 2013).

### 2.3 Stressors

The space use patterns of each individual whale were assessed relative to the spatial extent of four static (distance from two ports, distance from an effluent discharge site, area of whale watching) and two dynamic (commercial Dungeness crab pots, recreational fishing) anthropogenic stressors to estimate relative individual stressor exposure. Data layers for these anthropogenic stressors were compiled from multiple sources and classified as either static or dynamic based on whether the data varied temporally (Table 1). All stressor layers were scaled to be between 0 and 1 by dividing all values by the maximum values, so that 1 indicates high relative stressor intensity and 0 indicates no relative exposure.

**TABLE 1:** Summary of stressor data assessed relative to PCFG gray whale space use.

Stressor	Type	Temporal scale	Spatial scale	Location or source
Distance from Depoe Bay port	Static	—	1 km	44°48′33″ N, 124°4′13″ W
Distance from Newport port	Static	—	1 km	44°36′37″ N, 124°4′55″ W
Distance from effluent discharge site	Static	—	1 km	44°38′24″ N, 124°4′39″ W
Area of whale watching	Static	—	1 km	Whale Research EcoExcursions
Commercial Dungeness crab pots	Dynamic	Monthly (June–August)	5 km	Oregon Department of Fish and Wildlife
Recreational fishing	Dynamic	Monthly (June–October)	Size of fishing zones: 216, 260, 498 km^2^	Oregon Department of Fish and Wildlife

The marine ports of Newport and Depoe Bay lie within our study area and are trafficked with commercial and recreational activities, including fishing, crabbing, and whale watching. An effluent discharge line from a nearby pulp mill (Georgia‐Pacific Toledo) is located within the study area and is a point source for pollutants. These anthropogenic activities were identified as potential stressors to gray whales given evidence of behavioral and physiological responses by this group of whales to vessel traffic and ocean noise (Sullivan and Torres 2018; Lemos et al. 2022; Pirotta et al. 2023), documented entanglements with fishing gear (Scordino et al. 2020; Walsh et al. 2024), and high levels of exposure to pollutants (Torres et al. 2023) which may have an immunosuppressant effect on cetaceans (Marsili et al. 2012).

It was not possible to obtain direct data for all stressors, and we therefore had to rely on proxies of their variable intensity in space. Spatially explicit data layers on the intensity of boat traffic or the discharge of effluent do not exist for our study region. Therefore, we rely on the distance to each of these stressors as proxies for stressor exposure, with the assumption that with greater proximity to the source, each whale will have greater exposure (Cooke et al. 2020; Quintana et al. 2022). Increased proximity to a port represents increased vessel strike risk and noise exposure based on documented results in our study area that noise levels are higher closer to ports (Haver et al. 2023) and elevated noise levels are correlated with increased vessel counts (Lemos et al. 2022; Pirotta et al. 2023) in our study area. The Georgia‐Pacific Toledo effluent discharge site is a point source for potential contaminants (e.g., cyanide, metals) and releases ~11 million gallons of effluent per day in our study area (CH2MHill 2010). Thus, we calculated the distance from this discharge location across our study area as a proxy for pollutant load exposure, whereby increased proximity to the discharge site represents increased pollutant exposure. Depoe Bay has an active whale watching industry with ~12 boats operating tours daily, while Newport has one boat that conducts whale watching operations. Gray whales in this study system reduce their search effort for food when vessels are within 250 m (Sullivan and Torres 2018), indicating that whale watching is a potential stressor of concern to these whales. Whale watching varies greatly depending on weather conditions and spatial distribution of whales. The whale watching operators do not collect survey effort data and do not operate under any regulations limiting their effort in time or space. We collaborated with one Depoe Bay operator (Whale Research EcoExcursions) to compile their spatially explicit whale sighting data from 2016 to 2023 (excl. 2019 due to missing data) to broadly describe the spatial distribution of whale watching. We aggregated all whale sightings across years and created a single layer for the spatial distribution of whale watching, rather than yearly distributions, as an annual approach would be confounded by annual whale space use (i.e., whale watch vessels go to where the whales are) and cause bias in our analysis (Figure S2). Thus, the aggregated spatial extent represents an area of constant intensity of whale watching effort across all years, and we assume that with greater proximity to this area, each whale will have increased exposure to this potential stressor. We obtained temporally dynamic data for the two fishing effort stressors: commercial Dungeness crab fishing and recreational fishing effort, which were provided by the Oregon Department of Fish and Wildlife. Commercial Dungeness crab fishing effort was provided as the number of pots in the water for 2016–2022 (data for 2023 were not available). Recreational fishing data was provided as the number of permitted vessels for 2016–2023, which included charter and private vessels but excluded commercial fishing, research, and government vessels (see Texts S3 and S4 and Figure S3). All stressor data were summarized at the 55 latitudinal bin scale to match the scale of gray whale spatial encounters. Since the temporal scale of the SECR model was annual, we summarized mean dynamic stressor values across months within each year and confirmed no apparent temporal difference between months (Figures S4–S7).

### 2.4 Space Use and Exposure to Stressors

The activity center and range of individual *i* in year *t* correspond to the mean and variance of a normal distribution that defines that animal's space use and stressor exposure.

To estimate space use, at each MCMC chain iteration we calculated the relative occurrence of an individual in a latitude bin as the cumulative density of the normal distribution, defined by its activity center and range, between the boundaries of that bin, which were used as quantiles. We took the intercept of the baseline sighting rate for individual *i* in year *t*, *R*_*i,t*_, to reflect the time an individual spent in the study area over the course of the feeding season (i.e., its residency time). In practice, we multiplied the relative occurrence across latitude bins by *R*_*i,t*_, which we rescaled to be between 0 and 1, where 0 means completely absent from the study area and 1 means highest residency to the study area. The means of these weighted values across all iterations for individual *i* in year *t* were calculated for each latitude bin, resulting in one distribution of relative space use per individual in each year that accounted for the uncertainty around the activity center, activity range, and residency time.

To estimate an individual's exposure to each stressor within a given year, at each MCMC chain iteration, the relative occurrence of an individual in each latitude bin was multiplied by the scaled stressor value for the corresponding bin. We then summed the values for all latitude bins, resulting in one exposure value per iteration. Taken across all iterations, this formed a distribution of stressor exposure for each *i,t* combination. This distribution was then scaled by *R*_*i,t*_ as described above for the estimation of space use.

### 2.5 Post Hoc Results Exploration

We conducted post hoc explorations of the model‐derived space use and exposure estimates to explore ecological patterns. To discuss variation in activity range size across individuals and years, we used each individual's model‐derived activity range ($\sigma_{i,t}^{2}$) to calculate the individual's range (in km). The activity range is the $\sigma^{2}$ for a half normal distribution and so to describe the full normal distribution's 95% intervals, we multiply σ by 4. Finally, to convert this value to kilometers, we multiplied it by a conversion factor of 110 (1.1 ÷ 0.01) since the spatial scale of the model is latitude bins of 0.01°, which is equal to 1.1 km. To highlight variation in individual space use within and between years, we visually compared the space use of the six individuals with the highest interannual site fidelity. Additionally, we explored space use patterns of individuals by sex and age class. Sex was derived from photo‐identification catalogs (Marine Mammal Institute at Oregon State University and Cascadia Research Collective [Olympia, WA, USA]) and previous work (Lang et al. 2014; Lemos et al. 2020). Maturity (juvenile or adult) was assigned by age and length (Rice and Wolman 1971; Pirotta et al. 2024; Text S5). Wilcoxon rank‐sum tests were used to evaluate whether sex or maturity are related to activity range size (in km) since data were not normally distributed. To quantitatively test whether activity center location varies by age class, at each model iteration we calculated the mean of all juvenile and mature activity centers to produce a posterior distribution of mean activity centers for each group. We then compared these probabilistically by calculating the difference between the distributions assessing the proportion of the difference that overlapped with 0 (suggesting no difference between the two groups). Finally, to highlight the consequences of space use variation on individual stressor exposure, we visually compared the stressor exposure of three individuals with different space use patterns across 3 years.

## 3 Results

The number of surveys and total effort varied in space and time (Table 2, Figure 1). An average of 29 daily surveys were conducted with ~5200 km^2^ area surveyed each year. Greater survey effort did not necessarily equate to more total individuals seen within a year (e.g., 2020 and 2021), indicating that encounters were not solely driven by effort. On average, 60 marked individuals were encountered annually, but only ~20% met the criteria for model inclusion of being encountered on ≥ 5 survey days, though this varied between years (range: 4–27 individuals). A total of 55 unique individuals were included in the model, with variability in the number of years resighted (6 years = 1 individual, 5 = 4, 4 = 1, 3 = 10, 2 = 11, 1 = 28), resulting in 110 individual‐year model combinations. Sex and age composition of the 55 model‐included individuals was uneven (Sex: females = 27, males = 19, unknown = 9; Age: mature = 42, juvenile = 17 [note: four individuals matured from juvenile age during the course of the study period]).

**TABLE 2:** Summary of annual survey and individual gray whale sightings data used in the spatially explicit capture‐recapture (SECR) model: Photo‐identification surveys (No. surveys), area surveyed (Effort), total number of marked individuals identified (Total individuals), number of marked individuals included in the SECR model (Individuals included), and mean number (and range) of independent spatial encounters per individual included in the SECR model by year.

Year	No. surveys	Effort (km^2^)	Total individuals/Individuals included	Mean (range) spatial encounters per individual
2016	20	2636	63/4	8 (6–9)
2017	24	3584	68/8	10 (5–19)
2018	30	4116	77/27	9 (5–23)
2019	25	5761	62/15	9 (5–14)
2020	27	5461	42/6	9 (5–15)
2021	32	6391	37/7	13 (5–19)
2022	40	6393	61/23	13 (5–27)
2023	32	7284	71/20	12 (5–23)

**FIGURE 2:**
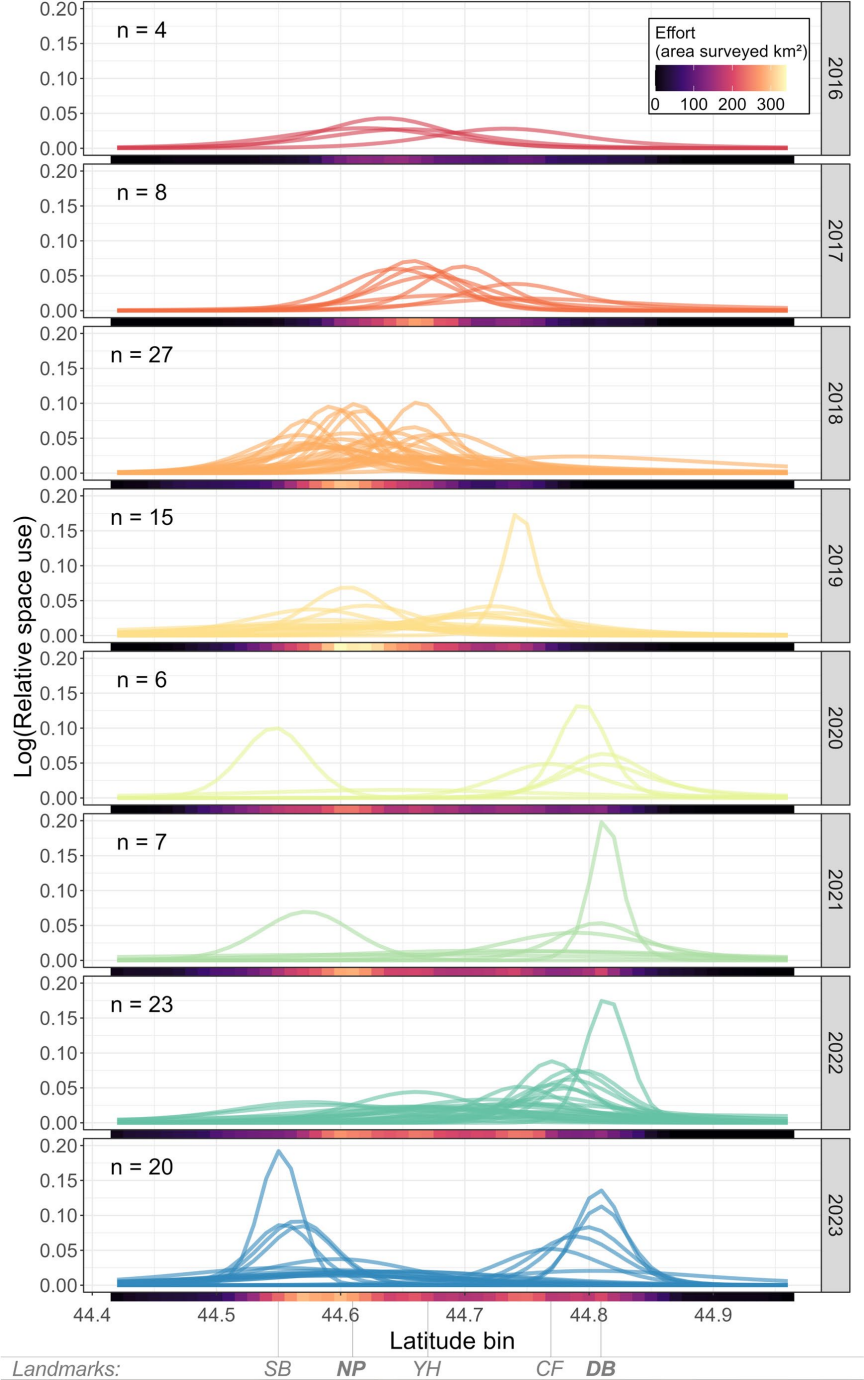
Relative space use (on log‐scale) of individual gray whales within the study area derived from the spatially explicit capture‐recapture model across the study period (2016–2023). Each line represents an individual. The total number of individuals is displayed in the top left corner of each panel. The amount of area surveyed (km^2^) as effort within each latitude bin is represented below each panel via a color ramp from no effort (black) to highest effort (yellow). The acronyms along the bottom correspond to spatial landmarks in Figure 1: CF, Cape Foulweather; DB, Depoe Bay; NP, Newport; SB, South Beach; YH, Yaquina Head.

Space use varied across time and by individual (Figure 2). From 2016 to 2018, individuals were more uniformly distributed in the middle of the study area (centered around latitude 44.65). Since 2019, space use distribution appears more bimodal, with one peak in the south (~44.55 latitude) and another peak in the north (~44.78 latitude), with less use of the central portion. However, this pattern may be due to more consistent survey effort in the north in the later years of the study. The mean activity range size across all individuals and years was 30.3 km (range = 6.30—108.5 km) (Table S3). Activity range size was not related to sex (Wilcoxon's test, *W* = 1160, *p* = 0.65) or age class (Wilcoxon's test, *W* = 1033, *p* = 0.34).

**FIGURE 3:**
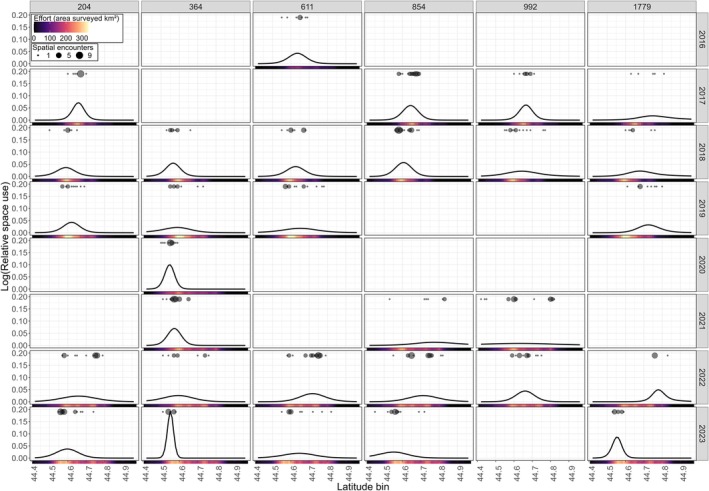
Relative space use (on log‐scale) within the study area derived from the spatially explicit capture‐recapture model for the six individual whales (identified using the individual code assigned by Cascadia Research Collective) seen in four or more years of the study period (an empty panel indicates the individual was not modeled for that year). Each vertical set of panels represents an individual's annual space use. Spatial encounter locations (black points) are scaled to represent the number of encounters. The amount of area surveyed (km^2^) as effort within each latitude bin is represented below each panel via a color ramp from no effort (black) to highest effort (yellow).

Variation in individual space use within and between years is exemplified for six individuals seen in ≥ 4 years (Figure 3), which reveals different site preferences and activity range sizes. While some individuals (e.g., #364, #611) show high fidelity to one area over many years, other individuals (e.g., #854, #1779) display variation in where their space use is centered between years. Furthermore, there are differences in the activity range sizes of individuals. For example, #364 typically had activity ranges smaller than 20 km, whereas other individuals had activity ranges greater than 40 km (e.g., #854, #992) (Table S3).

**FIGURE 4:**
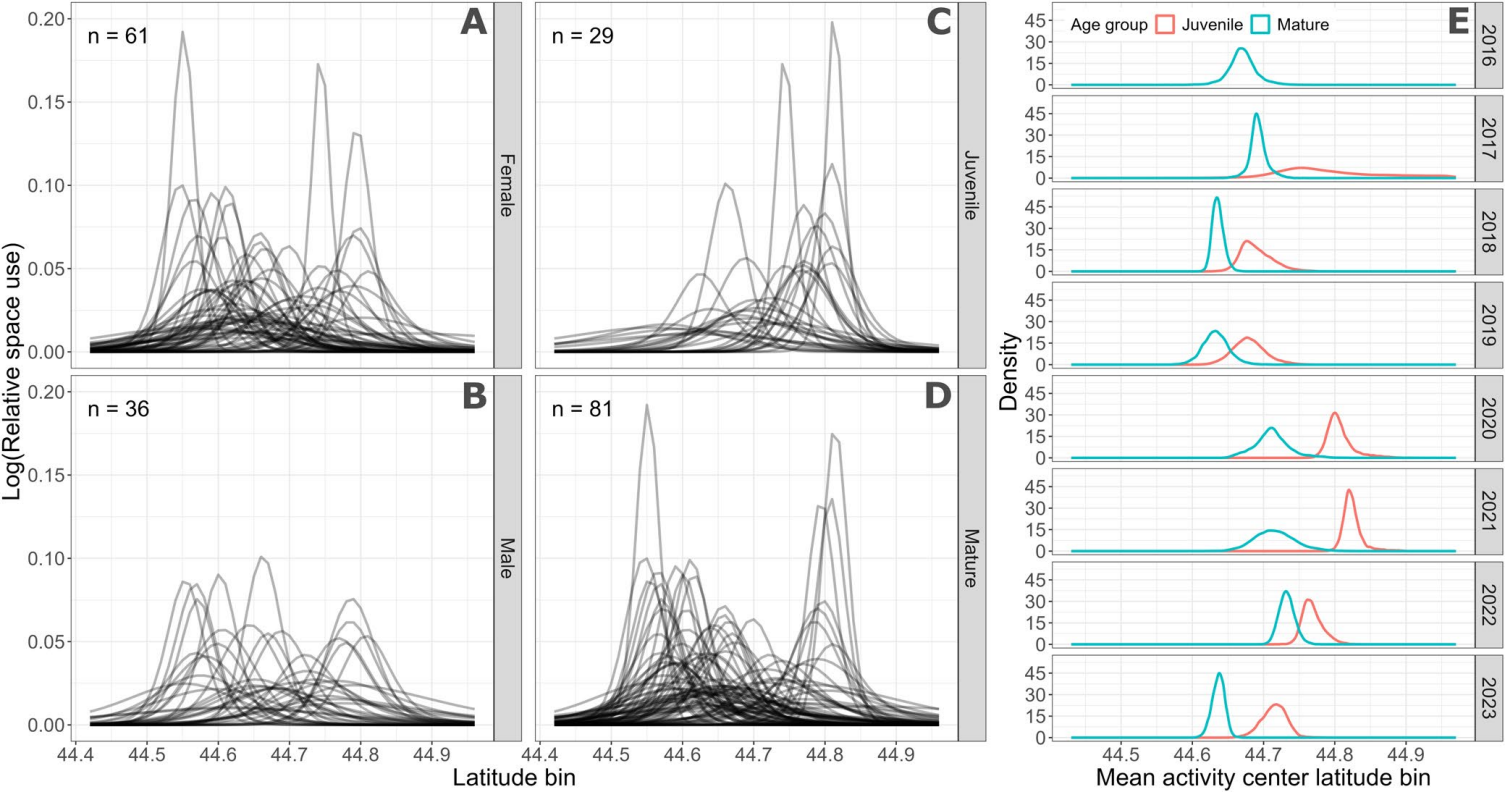
(A–D) Relative space use (on log‐scale) within the study area derived from the spatially explicit capture‐recapture (SECR) model of individual whales from different demographic groups: (A) Females, (B) Males, (C) Juveniles, and (D) Matures. Each line represents an individual‐year combination. Total number of individual‐year combinations are displayed in top left corner of each panel. Ten individuals had unknown sex and therefore their space use lines are not included in this figure. (E) Comparison of mean activity center between juvenile and mature individuals. Density curves derived from calculating the mean activity center for juveniles and matures at each iteration of the SECR model.

Sex does not appear to be associated with where individuals centered their space use (Figure 4A,B). Individuals of both sexes were distributed throughout the study area, without discernible preferences. Furthermore, both sexes contained individuals that showed high residency to one location with a small activity range (peaks on *y*‐axis), as well as individuals that had wide ranges (broad range on *x*‐axis), suggesting that there is no sex bias in the range of space use. While juveniles and mature whales were distributed across the study area (Figure 4C,D), the activity centers of juveniles were skewed to the north (Figure 4E). The difference in posterior distributions of mean activity centers between mature and juvenile individuals indicated a 98.7% probability that the mean of juvenile activity centers was more northerly than the mean of adult activity centers (Figure S8).

**FIGURE 5:**
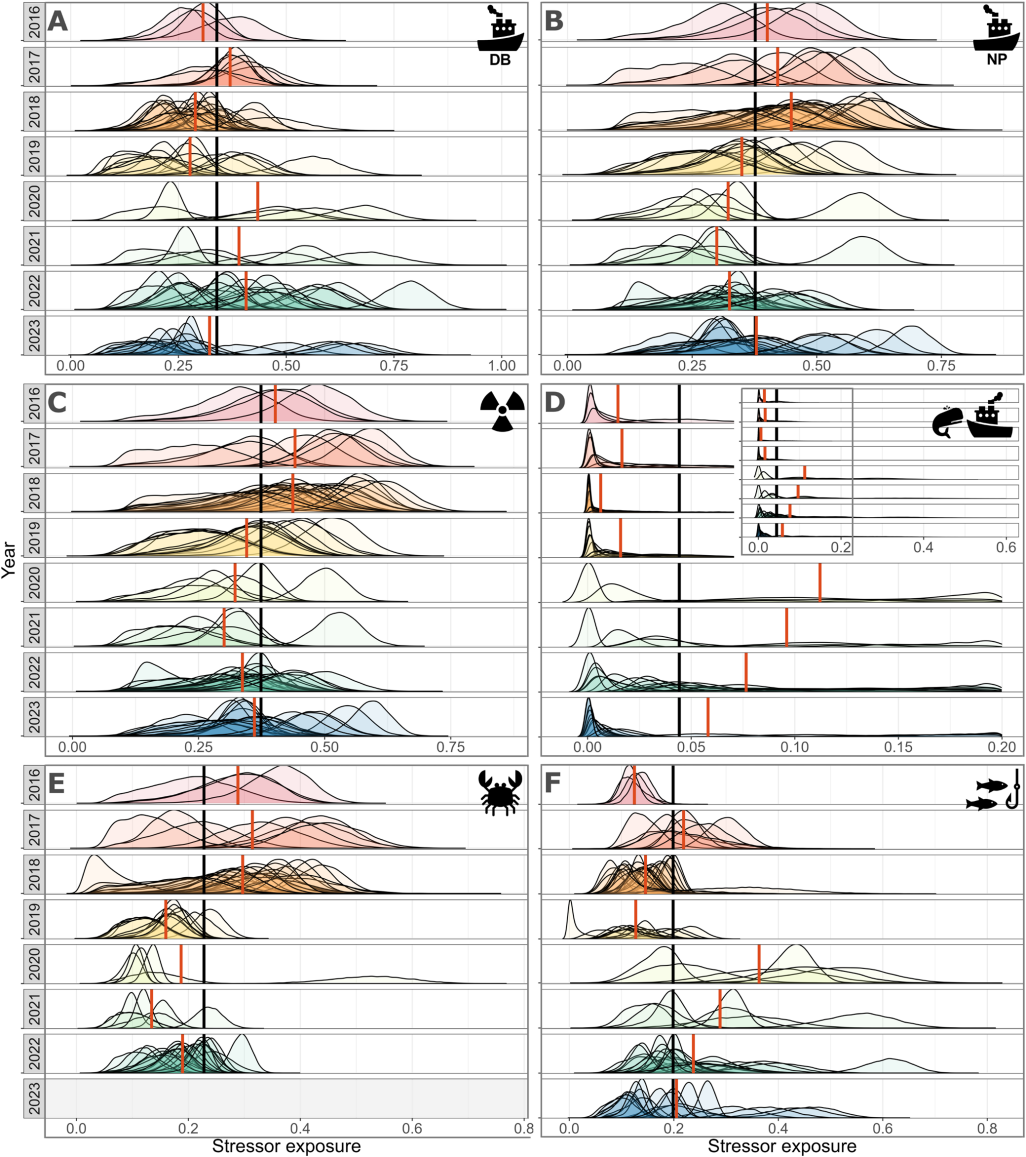
Individual gray whale exposure to (A) Depoe Bay port, (B) Newport port, (C) effluent discharge site, (D) whale watching, (E) commercial Dungeness crab pots, and (F) recreational fishing. Each line represents an individual's exposure calculated by multiplying the relative occurrence of an individual in each latitude bin by the scaled stressor value for the corresponding bin, and then summing the values for all latitude bins, resulting in one exposure value per iteration, which, summarized across all iterations, forms a distribution of stressor exposure for each individual. The black vertical lines represent the mean of all individual's median exposure to each stressor across years and the orange vertical lines represent the annual mean of all individual's median exposure to each stressor. For panel (D), the main plot shows the exposure to whale watching between exposure values 0 to 0.20 and the inset shows the full range of exposure values from 0 to 0.60.

Intraindividual variability in exposure to each stressor was high within and among years (Figure 5). Exposure to Depoe Bay and Newport ports shows contrasting trends when compared between years. In 2020–2022, annual mean exposure (orange line Figure 5A) to Depoe Bay port was higher than the mean exposure across all years (black line Figure 5A) due to the individual space use of many individuals being centered in the north (Figure 2). Resultantly, the opposite trend is realized for exposure to Newport port in these years (Figure 5B). However, one individual (#364) had relatively high exposure to Newport port in 2020 and 2021 relative to the other individuals in those years, since their space use was centered near Newport port (Figure 3). Annual mean exposure to the effluent discharge site was highest in 2017 (Figure 5C) but the annual mean exposures were close to the mean exposure across all years, likely because the effluent discharge site is located in the center of the study area (Figure 1). Most individuals had low exposure to whale watching, though some individuals had relatively high exposure (Figure 5D). Annual mean exposure was higher than the overall mean exposure to whale watching in 2020–2023. Exposure to commercial Dungeness crab pots appears to have reduced over time as annual mean exposures were higher than the overall mean exposure from 2016 to 2018, but have been lower than the overall mean exposure from 2019 onwards (Figure 5E). Annual mean exposure to recreational fishing was higher than the overall mean exposure in five of the eight study years, with especially high exposures of individuals in 2020–2021 (Figure 5F), which interestingly are the Covid‐19 pandemic years.

**FIGURE 6:**
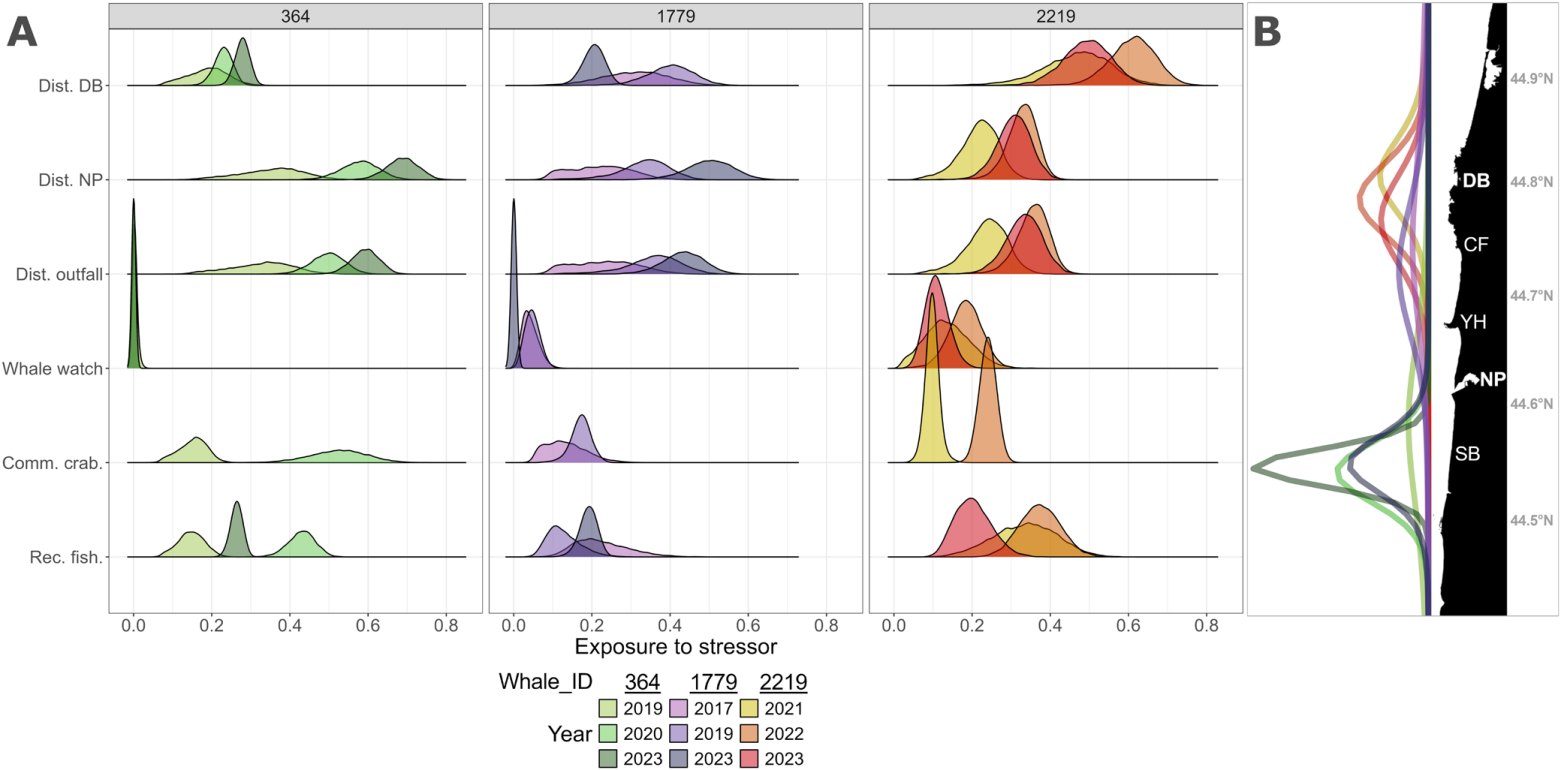
(A) Comparison of exposure to each stressor between three individual whales (identified using the individual code assigned by Cascadia Research Collective) with different space use patterns across 3 years. Each line represents an individual‐year combination. Stressor abbreviations: Comm. crab, commercial Dungeness crab fishing; Dist. DB, distance from Depoe Bay port; Dist. NP, distance from Newport port; Dist. outfall, distance from effluent discharge site; Rec. fish, recreational fishing; Whale watch, whale watching. (B) Space use patterns for each individual‐year combination represented in panel (A) derived from the spatially explicit capture‐recapture model. The colors in panel (B) correspond to the colors used in panel (A) for each individual‐year combination. The acronyms on the map correspond to spatial landmarks in Figure 1: CF, Cape Foulweather; DB, Depoe Bay; NP, Newport; SB, South Beach; YH, Yaquina Head.

Comparison of three individuals in three different years highlights the strong influence of individual space use on stressor exposure (Figure 6). Space use of individual #2219 was consistently concentrated in the north, leading to Depoe Bay port being the highest stressor this individual was exposed to and incurred some of the highest exposure to whale watching. Furthermore, #2219 had largely consistent exposure to all stressors across years due to the whale's space use fidelity between years. The same general consistency is seen for #364, with regular space use in the south, which leads to very high exposure to Newport port and low exposure to whale watching. However, exposure of #364 to the dynamic stressors (commercial Dungeness crab pots and recreational fishing) varied between years, likely due to spatiotemporal changes in stressor distribution and intensity. #1779 had variable space use patterns, which led to variable exposure to static stressors between years. #1779 had low exposure to whale watching in 2023 due to the whale's space use centered in the south, but exposure was higher in 2017 and 2019 as the whale's activity center was closer to the Depoe Bay port.

## 4 Discussion

We used a spatially explicit capture‐recapture model to quantify space use of individual gray whales and subsequently estimate their relative exposure to six anthropogenic stressors. The results revealed that exposure to stressors is highly variable across individuals and years given the heterogeneity of space use within the population and of stressor distribution (Figure 5), underscoring the complexity of managing wildlife populations. Juveniles exhibited a distributional bias to the north of our study region proximal to Depoe Bay port and where whale watching is centered. Thus, this age group, which is among the most sensitive within populations (Keen et al. 2021), experienced elevated exposure to these stressors. Furthermore, some individuals showed long‐term fidelity to specific locations, whereas other individuals showed large variation in space use patterns (Figure 3). Individuals occupied the entire latitudinal range of our study area, with different peaks in residency across years, though this may in part reflect variable spatial survey effort (Figure 2). Exposure to the six anthropogenic stressors was not predictable or consistent through time, making it impossible to identify one dominant stressor for the entire population. Thus, accounting for individual variation within a population appears critical to determine what proportion of the population is exposed to different stressors, and to what extent. Although many cetacean studies collect spatial data at the individual level, our study contributes to the relative paucity of studies that explore the role of individual differences in population space use patterns relative to a stressor with further consideration of the implications for population management (Christiansen et al. 2015; Pirotta et al. 2015; Glennie et al. 2021).

While several baleen whale species display site fidelity to feeding grounds across years at large scales (e.g., > 100 km^2^: Witteveen and Wynne 2017; Nichol et al. 2018; Ferreira et al. 2021), the high degree of fine‐scale and long‐term loyalty of individual whales to specific locations (Figure 3) has rarely been documented in a baleen whale species. Our model revealed 19 instances where individuals had activity ranges that spanned less than 15 km (Table S3) within our 60 km long study area. Similar fine‐scale (~20 km^2^) foraging ground site fidelity was documented from photo‐identification studies within the Western North Pacific gray whale population (Filatova et al. 2022), where two individuals observed across 3 years were sighted in the same locations each year, and among individual minke whales ( *Balaenoptera acutorostrata* ) feeding within the same subareas in two study sites (Monterey Bay, California and San Juan Islands, Washington, USA) across up to 9 years (Dorsey et al. 1990). Such fine‐scale spatial fidelity may be attributed to specialized foraging strategies (Dorsey et al. 1990; Bird et al. 2024), reliance on static, benthic features that are important for gray whale foraging (Filatova et al. 2022; Bird et al. 2024) or the influence of spatial memory (Abrahms et al. 2019). However, not all individuals in our study showed intra/interannual fidelity to a specific location, suggesting that individuals pursue different space use strategies to meet energetic needs.

We observed large variation in activity range size of individual whales, whereby the largest activity range (108.5 km) was more than 15 times larger than the smallest activity range (6.30 km), suggesting that different individuals may alter the size of their search radius to optimize energetic gains (Janetos 1982; Pyke 1984). Such differences in spatial strategies may be the result of differences in life‐history stage (Bixler and Gittleman 2000; van Beest et al. 2011), risk perception (Lima and Dill 1990), personality (Schirmer et al. 2019; Allegue et al. 2022) or resource availability (Svoboda et al. 2019). In fact, the number of individuals that met the criteria for model inclusion varied greatly across the study period, despite relatively equal amounts of effort (except for 2016 and 2017), suggesting that other factors, such as prey and environmental conditions, likely influenced space and strategy use. We note that our interpretation of activity range size variation as a spatial strategy derives from analysis of individuals that were selected based on higher fidelity (observed on ≥ 5 survey days in a year) to our relatively small study area. Individuals we describe as having large activity ranges may in fact have small activity ranges when considering their movements at a larger spatial scale across the entire PCFG feeding range (~1400 km). We therefore recommend that this SECR analysis be applied to spatial encounters of PCFG individuals across their entire feeding range, which would provide a more complete description of individual space use patterns and strategies, and the subsequent stressor exposure for this gray whale subgroup. While this effort would require comprehensively and repeatedly surveying a large area to obtain sufficient effort and sightings data to build the encounter histories necessary to inform individual space use across the entire range, collaboration between the network of scientists, managers, and whale watchers that already monitor this subgroup could enable a range‐wide SECR approach.

Individual differences in space use revealed that exposure to anthropogenic stressors among PCFG individuals was complex and nonuniform (Figures 5 and 6). While each of the stressors included in our analysis can affect the behavior and health of PCFG whales (Sullivan and Torres 2018; Lemos et al. 2022; Pirotta et al. 2023; Torres et al. 2023; Walsh et al. 2024), our study provides more highly resolved information on individual exposure levels within a portion of their feeding range. Such results are necessary to better establish mechanistic links between detecting behavioral or physiological responses in an individual and understanding whether these have long‐term impacts on vital rates (New et al. 2014; Pirotta et al. 2018, 2019). Whale watching in Iceland elicits behavioral responses in individual minke whales that lead to cessation of feeding activities and lost energy acquisition opportunities (Christiansen et al. 2013). However, implementation of a SECR model determined that whale watching does not pose a major threat, given the low overall exposure of individuals to this stressor (Christiansen et al. 2015). Hence, linking a known behavioral or physiological response to individual space use patterns relative to a stressor is critical to achieve realistic assessments of individual‐level disturbance impacts, which can then be scaled up to estimate population‐level consequences.

While our results provide insight into individual relative exposure to multiple stressors, exposure levels are not directly reflective of the behavioral, physiological, or health effects of each stressor on the whales. Information on the absolute intensity of these stressors and on their effects on individuals, in addition to our estimates of the variation in spatial overlap and resulting relative exposure levels, would be needed to assess the consequences of stressor exposure on individuals and on the population (Pirotta et al. 2018). Furthermore, the metrics we used to quantify the stressors could potentially have led to over‐ or underestimation of some exposure values. For example, while we evaluate exposure to boat traffic throughout our study area using distance from each harbor as a proxy, the boats leaving each harbor do not distribute equally over the entire study area. As a result, the range of influence of each harbor is probably restricted, and our exposure estimates using distance from harbor may be overestimated. Additionally, boat traffic and whale watching are not static stressors, and their static quantification in our analyses has likely led to underestimation of their intensity. For example, individual whales that have strong fidelity around Depoe Bay likely experience higher whale watching exposure than our results suggest, especially in years when an overall low number of whales are resident to the area causing whale watching activities to repeatedly target these whales (Figures 2, 5 and 6). Thus, spatiotemporally resolved boat counts and whale watching effort data would allow higher resolution of exposure quantification.

Our analysis highlighted that exposure can be highly heterogeneous over time (Figure 5), with some individuals experiencing greater stressor exposure relative to other individuals in the subgroup. Individual variation in relative exposure should be considered when assessing population‐level consequences and developing suitable management measures (Christiansen et al. 2015; Pirotta et al. 2021). We did not identify differences in space use (and thereby exposure) by sex (Figure 4). However, our analysis revealed that juveniles had a more northerly distribution of activity centers than adults (Figure 4E and Figure S10). While mature PCFG gray whales use stationary foraging tactics, juveniles employ a forward‐swimming foraging tactic in shallow habitat (Bird et al. 2024), suggesting that habitat (e.g., depth or benthic substrate) or prey aggregation characteristics in the north of our study area may be particularly suited for this tactic. Given this distributional bias, closer to the Depoe Bay port and where whale watching occurs, juveniles had some of the highest exposure values to these two stressors (e.g., individual #2219 in Figure 6). Furthermore, 55% and 69% of the juvenile individual‐year combinations had ≥ 50% of their exposure values distributed above the annual mean exposure for whale watching and distance to Depoe Bay port, respectively (Figure S9). Juveniles can be particularly vulnerable to disturbance events given their small body size, increased energetic needs due to growth, and naivety to risks (Keen et al. 2021). Elevated exposure to boat traffic could lead to lost feeding opportunities, with potential downstream consequences for their growth (Pirotta et al. 2024), maturation, reproduction (Pirotta et al. in press), and, ultimately, the population. Managers could consider imposing speed restrictions for vessels as they transit between recreational areas within this nearshore whale habitat and limitations on time spent with individual whales for whale watching boats. While population management plans inherently need to be implemented at a population level, the recognition and incorporation of variation in risk among individuals and demographic groups can improve regulation efficacy (Mimura et al. 2017).

SECR models are an effective way to explore individual space use and anthropogenic stressor exposure, yet appear underutilized in this regard, particularly in marine systems (but see Christiansen et al. 2015; Pirotta et al. 2015; Glennie et al. 2021). Their implementation in our system required us to relax the assumption of a closed population (Borchers and Efford 2008; Efford et al. 2009) because individuals move in and out of our study area during the feeding season. While violating this assumption prevents us from producing density estimates, our aim was to quantify where individual whales occur when in central Oregon. We restricted the model to assign an individual's activity center within our study area, and therefore the results do not represent an individual's space use for the entire PCFG feeding range. We originally intended for parameter *A* to represent the proportion of the year an individual spent in our study area, but it separated individuals into those that were not seen at all versus those that were. Hence, we used the intercept of the baseline sighting rate, *R*_*i,t*_, to capture individual residency instead. We assumed an exponential relationship between residency and number of encounters, since we allowed for multiple individual encounters within a day, which we expected to increase more than linearly with increasing survey effort. Therefore, we used *R*_*i,t*_ to scale an individual's space use, rather than log(*R*_*i,t*_). The annual temporal scale of our model means that any intraseasonal exposure patterns may have been lost. For example, an individual with five spatially explicit encounters in 1 month will experience different exposure to a dynamic stressor than an individual with five spatial encounters spread across 5 months; yet, these whales are considered identical in our model. We also summarized the dynamic stressors at an annual scale and while we did not identify clear differences in monthly stressor values (Figures S4 & S6), finer variation may still exist. Future work could explore the implications of our inclusion threshold (individuals seen on ≥ 5 days in a year) with a simulation study. Additionally, some estimated activity centers may not be ecologically meaningful if individuals have multiple activity centers. In this case, the model would either estimate an average activity center somewhere in between and assign a wide activity range, or fail to converge. The activity center parameter converged well; hence, the former is more likely to have occurred (e.g., individual #204 in 2022 and 2023 in Figure 3). Finally, our limited spatial encounter sample size prevented us from adding further complexity to the model. For example, space use strategy assignment based on activity range size could be built in to be estimated in the model. Furthermore, instead of using random effects around population means for some parameters (e.g., activity range), with a larger sample size the model could be configured to estimate individual means across years, around which year‐specific parameters would then be distributed.

While our results showed variable individual space use patterns, we did not investigate the drivers of these patterns. Knowledge of the underlying mechanisms driving this heterogeneity would further support the development of effective and anticipatory management plans. Given that our study took place on a foraging ground, spatiotemporal resource availability may have fluctuated between years, leading to varying site fidelity, space use patterns, and strategies. In fact, during the study period, declines in gray whale prey were documented in southern Oregon, associated with declines in kelp health in response to a rise in sea urchin occurrence and a marine heatwave (Hildebrand et al. 2024). Therefore, future investigations should explore the environmental and oceanographic drivers of individual whale occurrence within our study region. We also did not examine the behavioral or physiological consequences of variable stressor exposure levels on individuals; yet, given our finding that juveniles display a distributional bias to a more exposed area, such an examination is needed and could elucidate trade‐offs of different space use strategies. Establishing the link between space use, exposure, and consequences is critical to more accurately quantify the impact of multiple stressors on the population overall.

## Author Contributions

**Lisa Hildebrand:** conceptualization (equal), data curation (equal), formal analysis (lead), investigation (equal), methodology (lead), visualization (lead), writing – original draft (lead), writing – review and editing (lead). **Leslie New:** formal analysis (supporting), funding acquisition (supporting), methodology (supporting), writing – review and editing (equal). **Enrico Pirotta:** formal analysis (supporting), methodology (supporting), writing – review and editing (equal). **Joshua D. Stewart:** conceptualization (supporting), formal analysis (supporting), methodology (supporting), writing – review and editing (equal). **Ines Hildebrand:** data curation (equal), writing – review and editing (equal). **Carrie Newell:** data curation (equal), investigation (equal), writing – review and editing (equal). **K. C. Bierlich:** investigation (equal), writing – review and editing (equal). **Clara N. Bird:** investigation (equal), writing – review and editing (equal). **Alejandro Fernandez Ajó:** investigation (equal), writing – review and editing (equal). **Daniel Turek:** formal analysis (supporting), methodology (supporting), writing – review and editing (equal). **Leigh G. Torres:** conceptualization (equal), data curation (equal), funding acquisition (lead), investigation (lead), project administration (lead), supervision (lead), writing – original draft (supporting), writing – review and editing (equal).

## Conflicts of Interest

The authors declare no conflicts of interest.

## Supporting information


Data S1.

## Data Availability

Data and code are provided at the Figshare data repository: https://figshare.com/s/11a55018a21660259c73.
