# Supplementary material for: Bayesian Estimation of Individual Gray Whale Space Use Reveals Differential Exposure to Stressors
Source: Ecol Evol. 2025 May 25;15(5):e71330. doi: 10.1002/ece3.71330 (PMC12104204; doi:10.1002/ece3.71330)
Supplement: Supplementary file 1 — Data S1. [file ECE3-15-e71330-s001.docx]

**Supplemental material:**

**Bayesian estimation of individual gray whale space use reveals differential exposure to stressors**

Table of Contents

[Text S1: Quantification of spatial survey effort from continuous observation conditions and survey tracklines. 2](#_Toc181008519)

[Table S1. Rubric used to assign a sightability category to each segment of the daily survey tracklines based on continuous observations of Beaufort Sea State and swell height. 2](#_Toc181008520)

[Table S2. Prior distribution of all parameters 2](#_Toc181008521)

[Text S2: Prior sensitivity analysis and posterior distributions 3](#_Toc181008522)

[Comparison of spatial distributions of whale watch sightings (Figure S2) 4](#_Toc181008523)

[Data collection methods by Oregon Department of Fish and Wildlife for dynamic stressors (Texts S3-4) 5](#_Toc181008524)

[Temporal variation in dynamic stressors (Figures S4-7) 8](#_Toc181008525)

[Text S5: Details for sex and age determination of individual Pacific Coast Feeding Group gray whales 10](#_Toc181008526)

[Results of activity range summaries (Table S3) 10](#_Toc181008527)

[Results of activity center analysis by age group (Figures S8-9) 15](#_Toc181008528)

[References 16](#_Toc181008529)

# *Text S1: Quantification of spatial survey effort from continuous observation conditions and survey tracklines.*

Observation conditions (Beaufort Sea State [BSS], swell height, visibility) were continuously recorded during survey effort and GPS boat tracklines were stored at the end of each survey day. To summarize spatial survey effort (km^2^), observation conditions were used to assign a categorical metric of sightability (ranging from poor to excellent) to each survey segment of the trackline based on a rubric (Table S1) derived from experienced field observations. The sightability categories were then converted to a survey effort distance from trackline (poor = 0.5 km; fair = 1 km; good = 2 km; excellent = 3 km) derived from experienced field observations and literature (Barlow et al., 2024). If visibility observations indicated that fog, rain and/or marine haze limited survey effort distance, then the distance value recorded by the field team was assigned instead of the sightability category that would have been assigned based on BSS and swell height. These survey effort distances were applied as a buffer to their corresponding survey segment of the tracklines, resulting in the total amount of area surveyed (km^2^) per survey segment. The total amount of area surveyed was then summed within each latitude bin for the year.

# *Table S1. Rubric used to assign a sightability category to each segment of the daily survey tracklines based on continuous observations of Beaufort Sea State and swell height.*

| **Swell Height (ft)** | **Beaufort Sea State** | | | |
| --- | --- | --- | --- | --- |
|  | 0-1 | 2 | 3 | 4 |
| ≤ 3 | Excellent | Good | Fair | Fair |
| 4 | Excellent | Good | Fair | Poor |
| 5 | Good | Fair | Poor | Poor |
| ≥ 6 | Fair | Poor | Poor | Poor |

# *Table S2. Prior distribution of all parameters*

| **Parameter** | **Symbol** | **Prior** |
| --- | --- | --- |
|  |  |  |
| Population mean for activity range | $\mu$ | *Normal*(-3, 1) |
| Population variance for activity range | ω | *Uniform*(0, 5) |
| Effort slope | $\beta_{e}$ | *Normal*(0, 0.1) |
| Intercept of the baseline sighting rate for individual *i* in year *t* | $R_{i,t}$ | *Normal*(0, 1) |
| Activity range random effect for individual *i* and year *t* | $M_{i,t}$ | *Normal*(0, ω) |
| Activity center for individuals estimated to be inside study area | $C_{i,t}$ | *Uniform*(-0.27, 0.27) |

# *Text S2: Prior sensitivity analysis and posterior distributions*

We conducted a prior sensitivity analysis for the population-level parameters in our spatially explicit capture-recapture model, namely the population mean for activity range ($\mu$), the population variance for activity range (ω) and the effort slope ($\beta_{e}$). We increased the standard deviation for each of the priors by multiplying the original standard deviation by 10. Based on this analysis (Figure S1), we believe that sensitivity to prior specification was low for these three population-level parameters.

The posterior 2.5^th^, 50^th^ (median) and 97.5^th^ quartiles for these three parameters are as follows:

$\mu$ = -6.25, -5.98, -5.71; ω = 1.00, 1.20, 1.47; $\beta_{e}$ = 0.07, 0.10, 0.13.


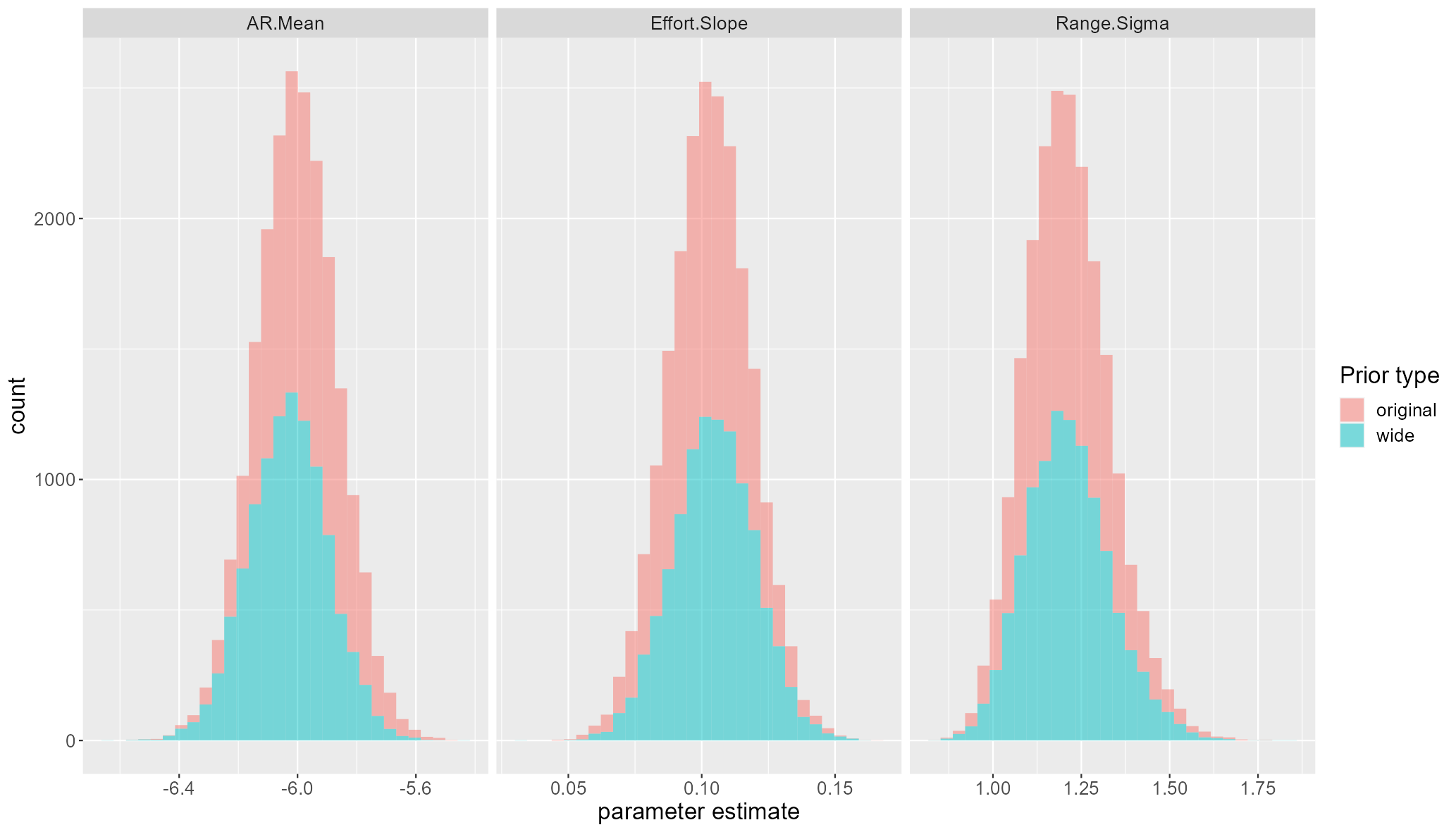


*Figure S1. Comparison of original and wide priors for the population-level parameters included in the spatially explicit capture-recapture model. AR.Mean = population mean for activity range (*$\mu$*); Range.Sigma = the population variance for activity range (*ω*); Effort.Slope = effort slope (*$\beta_{e}$*).*

# Comparison of spatial distributions of whale watch sightings


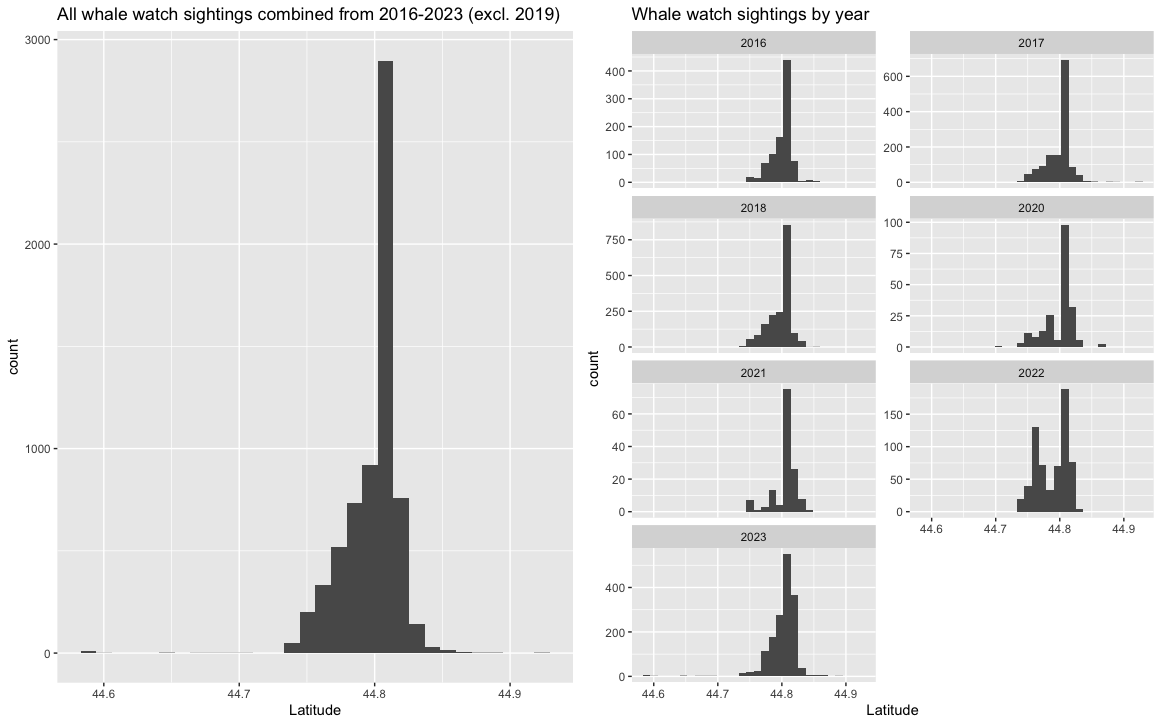


*Figure S2. Distribution of latitudinal location of all whale watch sightings combined for 2016-2023 (excluding 2019 due to missing data; left panel). Distribution of latitudinal location of whale watch sightings by year for 2016-2023 (right panel).*

# Data collection methods by Oregon Department of Fish and Wildlife for dynamic stressors

*Text S3: Methodology for deriving Dungeness crab fishing estimates by the Oregon Department of Fish and Wildlife. Adapted from Derville et al., 2023.*

Commercial Dungeness crab fishing data were provided by the Oregon Department of Fish and Wildlife (ODFW). The raw data are sourced from logbook data that are self-reported by fishing vessel captains. Logbooks from both Oregon and Washington were combined and processed together as fishing vessels from both states fish off Oregon. ODFW implemented several data quality filters to remove incomplete or inaccurate data. Series of individual pots are typically set in a line, usually along a depth contour, termed a “string”. Each string represents a logbook record, which includes information about the vessel, port, date, depth, soak time, number of pots pulled, and start and end location. ODFW removed all records with missing or incomplete data on location, pot pulls, date, port, or vessel identification. ODFW also removed records with obviously erroneous location data (e.g., points on land or beyond the depth of the fishery), more than 160 pot pulls, the start and end location, or a distance greater than 28 km between the start and end locations. ODFW retained records with incomplete or inaccurate depth or soak time as these fields were not ultimately used for estimates. After applying these filters to all data, 92% of the raw data were retained.

After filtering for data quality, ODFW allocated each vessel’s pot limit (the maximum number of pots that may legally be set at one time) proportional to pot pulls across fishing locations that vessel recorded in its logbooks, at a monthly scale, to generate estimates of the number of pots in the water for each 5 km grid cell for vessels that appeared in logbook data.

Logbook data were sometimes incomplete, either due to 30% subsampling for data entry in some fishing seasons, non-compliance (landings with no logbook submitted), and logbook records missing critical data fields. In these instances, ODFW completed a series of steps to estimate the maximum potential number of pots per grid cell. First, for each year and month combination, ODFW divided the sum of pot limits across vessels represented in the processed logbook data by the sum of pot limits across vessels that made a landing to derive the percentage of potential pots represented in logbook data. The estimates of pots per grid cell from logbook data were then divided by this percentage for each year x month x 5 km grid cell to scale estimates to the total potential pots deployed:

$$Number of pots deployed= \frac{z}{m/n}$$

Where *z* is the sum of pots allocated to a grid cell for vessels represented in logbook data, *m* is the sum of pot limits for vessels represented in the logbook data, and *n* is the sum of pot limits for all vessels that made a landing.

This approach for estimating maximum potential number of pots per grid cell in instances of incomplete data is based on several assumptions, including that any time a vessel has made a landing within a year x month: 1) the vessel’s entire pot limits (200, 300, or 500 pots) was deployed in the water for the entire month, 2) the vessel’s pot limit is distributed spatially in proportion to pot pulls, and 3) the spatial distribution of pot limits for vessels that are not represented in the logbook is the same as for vessels that are. Months that were closed to fishing or where fishing did not start due to negotiations for price for more than 15 days were removed from the fishing estimates.

*Text S4: Methodology for deriving recreational fishing estimates from the Oregon Department of Fish and Wildlife.*

Recreational fishing data were provided by the Oregon Department of Fish and Wildlife (ODFW). ODFW conducts angler interviews at ports throughout the state, including in Newport and Depoe Bay. Upon returning to port, anglers are asked a series of questions pertaining to their fishing trip, including the type of fish that were targeted, number of anglers on the boat, what time they launched to go fishing, and anglers are asked to indicate on a map where they spent the majority of their time fishing. This spatial information is categorized into categorical reef locations (see reefs 17-22 in Figure S3). Vessel types included in these counts were charter and private vessels, and did not include commercial fishing, research, or government vessels. ODFW interviewers have a minimum goal to interview 20% of all vessels that return to port. Thus, while the recreational fishing data do not include every vessel that returns to port, the ≥ 20% that are interviewed are assumed to be a representative sample.

For our analysis, we only included vessels that indicated they spent the majority of their time in inshore reef locations 18, 20 and 22, since these encompass the latitudinal range of our study area and we do not have spatial captures of gray whales beyond the 30-fathom regulatory line (dotted red line on Figure S3).

*Figure S3. Map used by Oregon Department of Fish and Wildlife while conducting angler interviews to locate which reef zones anglers fished within.*

# Temporal variation in dynamic stressors


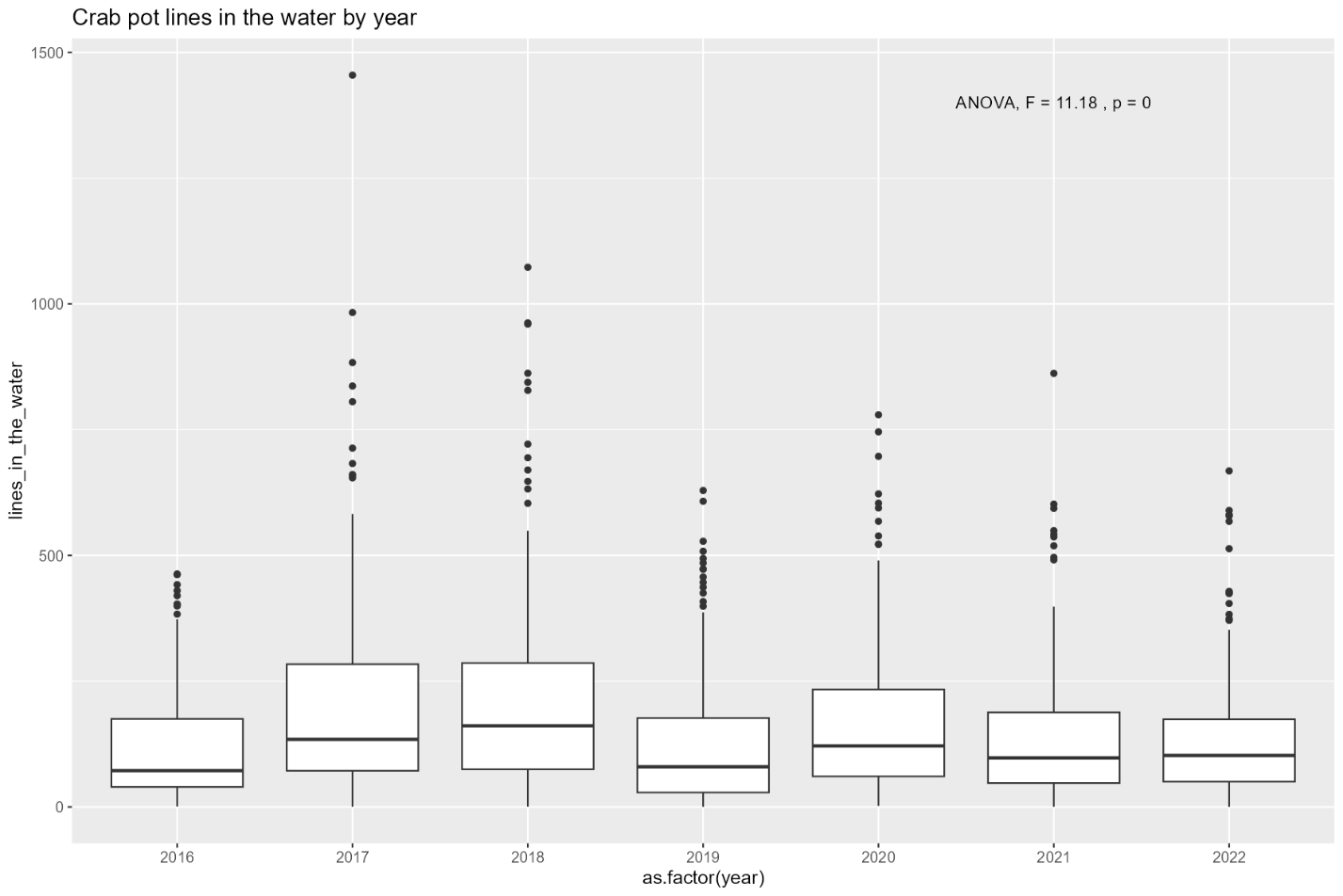


*Figure S4. Comparison of commercial Dungeness crab pot lines in the water between years (2016-2022).*


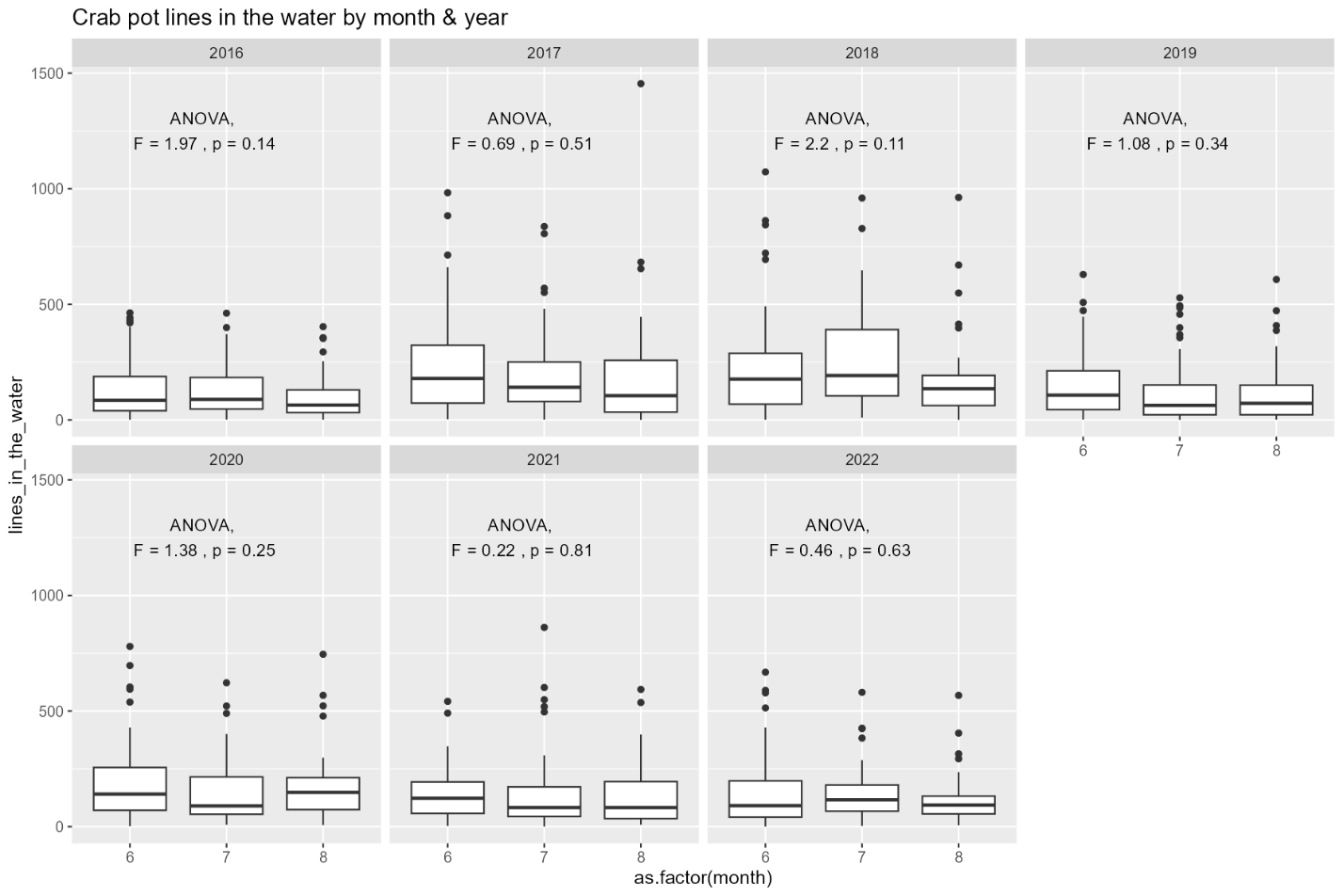


*Figure S5. Comparison of commercial Dungeness crab pot lines in the water between months (June-August) within years (2016-2022).*


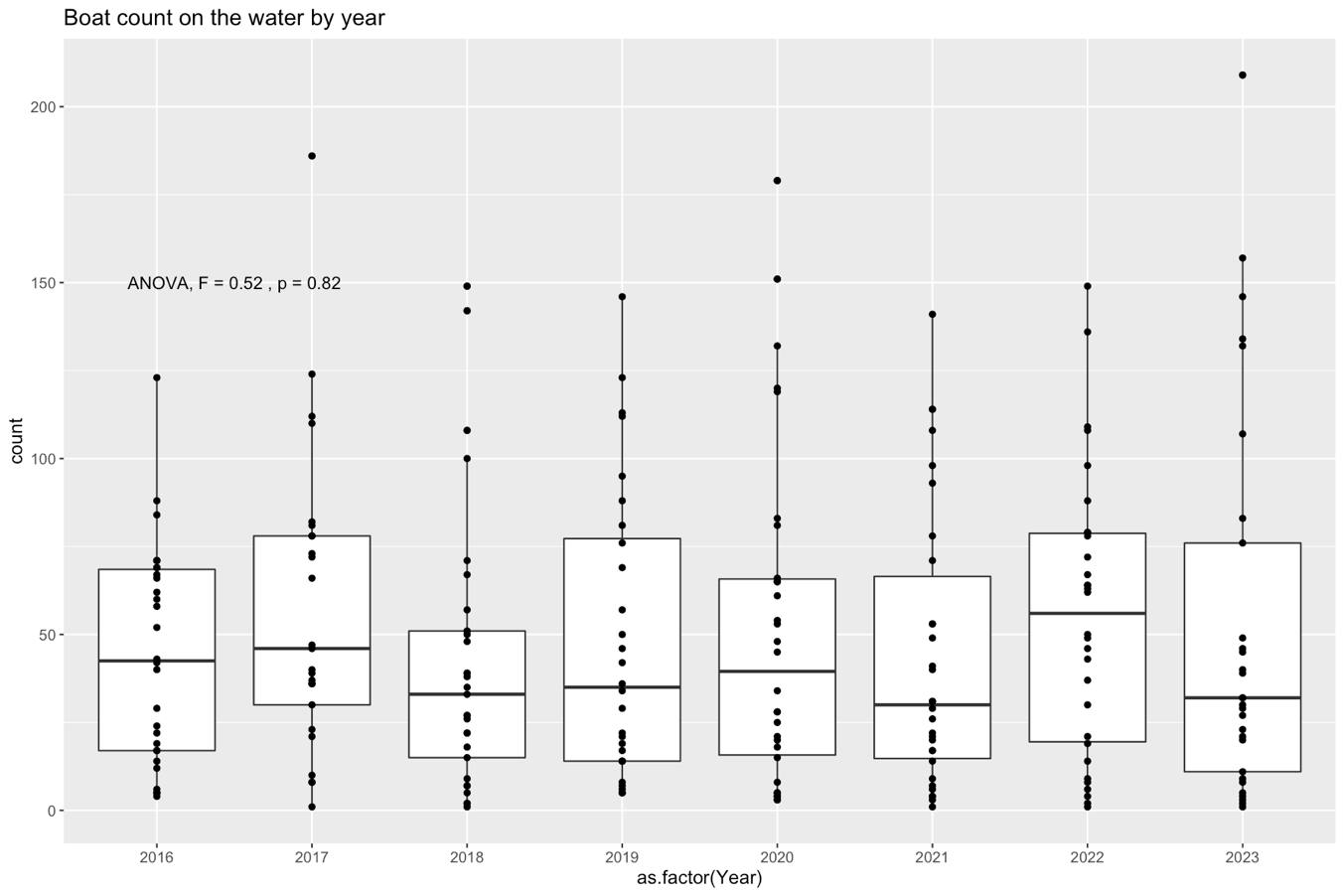


*Figure S6. Comparison of recreational fishing vessel counts between years (2016-2023).*


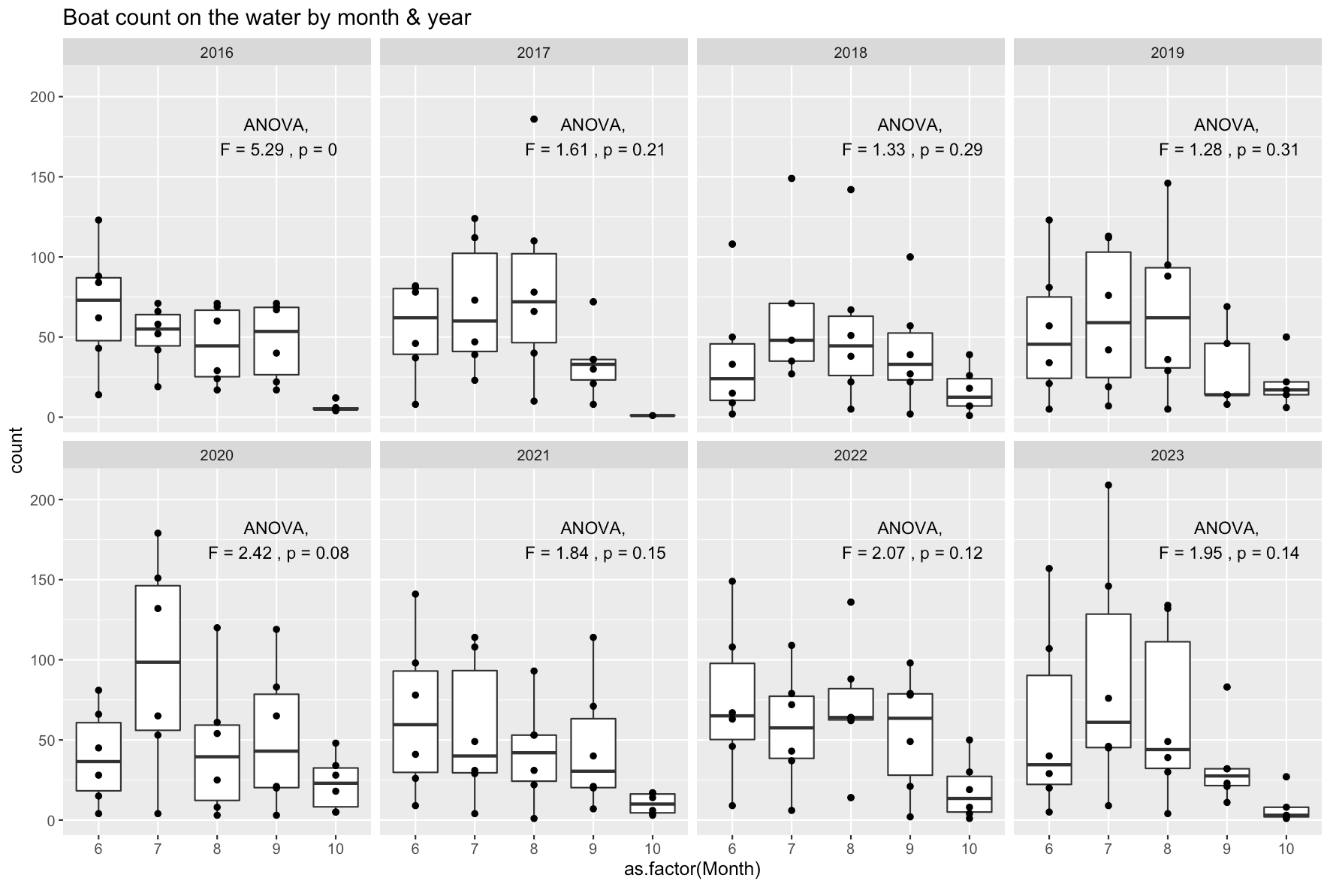


*Figure S7. Comparison of recreational fishing vessel count between months (June-October) within years (2016-2023).*

# Text S5: Details for sex and age determination of individual Pacific Coast Feeding Group gray whales

Sex was derived from existing information in photo-identification catalogs held by the Marine Mammal Institute at Oregon State University and Cascadia Research Collective (Olympia, WA, USA), previous genetic analyses (Lang et al., 2014), or genetic analyses of fecal samples (Soledade Lemos et al., 2020). For age class, we classified individuals with a known age or minimum age ≥ 8 years as mature (mean age of sexual maturity (Rice & Wolman, 1971)), whereas individuals with a known age < 8 years were classified as juvenile. For individuals with a minimum age < 8 years, we determined their age using their length derived from an individual growth model (Pirotta et al., 2024).

# Results of activity range summaries

*Table S3. Mean and standard deviation range (km) of individuals Individuals (ID) are identified using the individual code assigned by Cascadia Research Collective (Olympia, WA, USA).*

| **ID** | **Year** | **Age group** | **Mean range (km)** | **SD range (km)** | **Number of spatial encounters** |
| --- | --- | --- | --- | --- | --- |
| 169 | 2016 | Mature | 32.5 | 14.15 | 8 |
| 611 | 2016 | Mature | 23.4 | 7.73 | 9 |
| 206 | 2016 | Mature | 33.2 | 15.57 | 7 |
| 308 | 2016 | Mature | 32.3 | 16.05 | 6 |
| 1779 | 2017 | Juvenile | 44.4 | 23.9 | 5 |
| 992 | 2017 | Mature | 17.1 | 5.29 | 11 |
| 854 | 2017 | Mature | 19.5 | 4.46 | 19 |
| 295 | 2017 | Mature | 40.5 | 24.08 | 7 |
| 698 | 2017 | Mature | 22 | 6.41 | 15 |
| 308 | 2017 | Mature | 25.7 | 13 | 7 |
| 94 | 2017 | Mature | 16.3 | 6.7 | 8 |
| 204 | 2017 | Mature | 15.2 | 4.57 | 11 |
| 537 | 2018 | Mature | 21.4 | 10.95 | 6 |
| 878 | 2018 | Mature | 11.7 | 3.21 | 13 |
| 1779 | 2018 | Juvenile | 40.1 | 20.92 | 7 |
| 900 | 2018 | Mature | 24.3 | 12.17 | 7 |
| 169 | 2018 | Mature | 11.9 | 4.07 | 9 |
| 992 | 2018 | Mature | 46.7 | 23.31 | 14 |
| 565 | 2018 | Mature | 12.3 | 3.12 | 14 |
| 254 | 2018 | Mature | 23.4 | 9.74 | 9 |
| 136 | 2018 | Mature | 19.9 | 7.64 | 9 |
| 278 | 2018 | Mature | 13.2 | 6.64 | 5 |
| 611 | 2018 | Mature | 24.6 | 9.62 | 11 |
| 192 | 2018 | Mature | 22.1 | 11.17 | 7 |
| 219 | 2018 | Mature | 12 | 4.41 | 9 |
| 854 | 2018 | Mature | 20.7 | 4.13 | 23 |
| 532 | 2018 | Mature | 44.1 | 24.82 | 6 |
| 860 | 2018 | Mature | 17.7 | 6.24 | 10 |
| 186 | 2018 | Mature | 37.5 | 21.07 | 7 |
| 1601 | 2018 | Juvenile | 19.4 | 12 | 5 |
| 1863 | 2018 | Juvenile | 34.8 | 19.22 | 5 |
| 813 | 2018 | Mature | 18 | 11.17 | 5 |
| 84 | 2018 | Mature | 37.5 | 12.37 | 7 |
| 1509 | 2018 | Mature | 15.6 | 7.05 | 7 |
| 1840 | 2018 | Juvenile | 11.7 | 2.84 | 12 |
| 204 | 2018 | Mature | 25.4 | 12.01 | 8 |
| 364 | 2018 | Mature | 19 | 6.46 | 10 |
| 1782 | 2018 | Juvenile | 32.4 | 19.94 | 5 |
| 2171 | 2018 | Juvenile | 19 | 7.79 | 9 |
| 1455 | 2019 | Juvenile | 58.9 | 31.39 | 7 |
| 878 | 2019 | Mature | 43.1 | 21.89 | 5 |
| 1779 | 2019 | Juvenile | 29.5 | 11.19 | 10 |
| 169 | 2019 | Mature | 25.6 | 9.49 | 11 |
| 565 | 2019 | Mature | 15.3 | 4.25 | 13 |
| 611 | 2019 | Mature | 51 | 26.1 | 14 |
| 701 | 2019 | Mature | 49.5 | 27.35 | 5 |
| 186 | 2019 | Mature | 21.7 | 9.84 | 6 |
| 1801 | 2019 | Juvenile | 36.6 | 15.26 | 13 |
| 2187 | 2019 | Juvenile | 6.9 | 3.27 | 6 |
| 204 | 2019 | Mature | 22.2 | 7.86 | 11 |
| 364 | 2019 | Mature | 39.2 | 16.65 | 10 |
| 1782 | 2019 | Juvenile | 56.5 | 24.2 | 11 |
| 2171 | 2019 | Juvenile | 74.4 | 35.16 | 11 |
| 1753 | 2019 | Juvenile | 28 | 13.11 | 7 |
| 565 | 2020 | Mature | 66.5 | 33.13 | 8 |
| 196 | 2020 | Mature | 20.7 | 10.25 | 5 |
| 1237 | 2020 | Mature | 9.4 | 2.85 | 10 |
| 1838 | 2020 | Juvenile | 20.3 | 8.17 | 7 |
| 2425 | 2020 | Juvenile | 17.8 | 7.72 | 8 |
| 364 | 2020 | Mature | 12.4 | 2.81 | 15 |
| 992 | 2021 | Mature | 108.5 | 47.28 | 19 |
| 196 | 2021 | Mature | 27.9 | 6.58 | 18 |
| 854 | 2021 | Mature | 55.8 | 22.41 | 7 |
| 1237 | 2021 | Mature | 66.6 | 26.89 | 17 |
| 2219 | 2021 | Juvenile | 17.8 | 7.92 | 5 |
| 2425 | 2021 | Juvenile | 6.3 | 2.77 | 7 |
| 364 | 2021 | Mature | 17 | 3.37 | 19 |
| 537 | 2022 | Mature | 30.8 | 10.64 | 10 |
| 2182 | 2022 | Juvenile | 12.9 | 4.25 | 10 |
| 1779 | 2022 | Juvenile | 19.8 | 10.62 | 5 |
| 992 | 2022 | Mature | 23.3 | 5.36 | 15 |
| 136 | 2022 | Mature | 35.2 | 17.47 | 7 |
| 611 | 2022 | Mature | 32.5 | 8.77 | 22 |
| 196 | 2022 | Mature | 15.6 | 6.76 | 9 |
| 854 | 2022 | Mature | 40.2 | 13.43 | 19 |
| 714 | 2022 | Mature | 51.4 | 19.29 | 9 |
| 860 | 2022 | Mature | 35.8 | 19.06 | 6 |
| 186 | 2022 | Mature | 40.7 | 12.39 | 21 |
| 1237 | 2022 | Mature | 85 | 23.81 | 17 |
| 1718 | 2022 | Mature | 32.2 | 13.8 | 8 |
| 308 | 2022 | Mature | 25.7 | 11.59 | 7 |
| 2187 | 2022 | Juvenile | 18.2 | 8.55 | 6 |
| 204 | 2022 | Mature | 46.3 | 15.16 | 27 |
| 2219 | 2022 | Juvenile | 15.9 | 4.79 | 14 |
| 2477 | 2022 | Juvenile | 18.7 | 8.67 | 7 |
| 89 | 2022 | Mature | 18.3 | 6.96 | 11 |
| 2218 | 2022 | Mature | 7.9 | 1.84 | 16 |
| 364 | 2022 | Mature | 39.1 | 11.67 | 18 |
| 297 | 2022 | Mature | 85.6 | 39.02 | 14 |
| 1753 | 2022 | Juvenile | 53.7 | 22.8 | 16 |
| 1455 | 2023 | Mature | 41.2 | 16.06 | 7 |
| 537 | 2023 | Mature | 13.1 | 3.73 | 12 |
| 2182 | 2023 | Juvenile | 57.4 | 22.38 | 11 |
| 1779 | 2023 | Mature | 12.4 | 4.05 | 9 |
| 1766 | 2023 | Mature | 54 | 20.07 | 9 |
| 2532 | 2023 | Juvenile | 40.4 | 17.27 | 5 |
| 1070 | 2023 | Mature | 38.1 | 16.16 | 7 |
| 1741 | 2023 | Mature | 42.3 | 12.94 | 11 |
| 611 | 2023 | Mature | 43.3 | 16.89 | 12 |
| 854 | 2023 | Mature | 38 | 11.39 | 14 |
| 2530 | 2023 | Juvenile | 14.2 | 3.3 | 14 |
| 204 | 2023 | Mature | 27.8 | 6.25 | 19 |
| 2219 | 2023 | Juvenile | 19.3 | 5.3 | 10 |
| 2477 | 2023 | Juvenile | 10.5 | 2.98 | 10 |
| 1108 | 2023 | Mature | 16.3 | 3.75 | 14 |
| 2218 | 2023 | Mature | 9.1 | 2.49 | 11 |
| 364 | 2023 | Mature | 7.4 | 1.34 | 23 |
| 1782 | 2023 | Mature | 13.6 | 2.58 | 22 |
| 297 | 2023 | Mature | 41.8 | 13.8 | 14 |
| 1753 | 2023 | Mature | 47 | 16.45 | 16 |

# Results of activity center analysis by age group

*
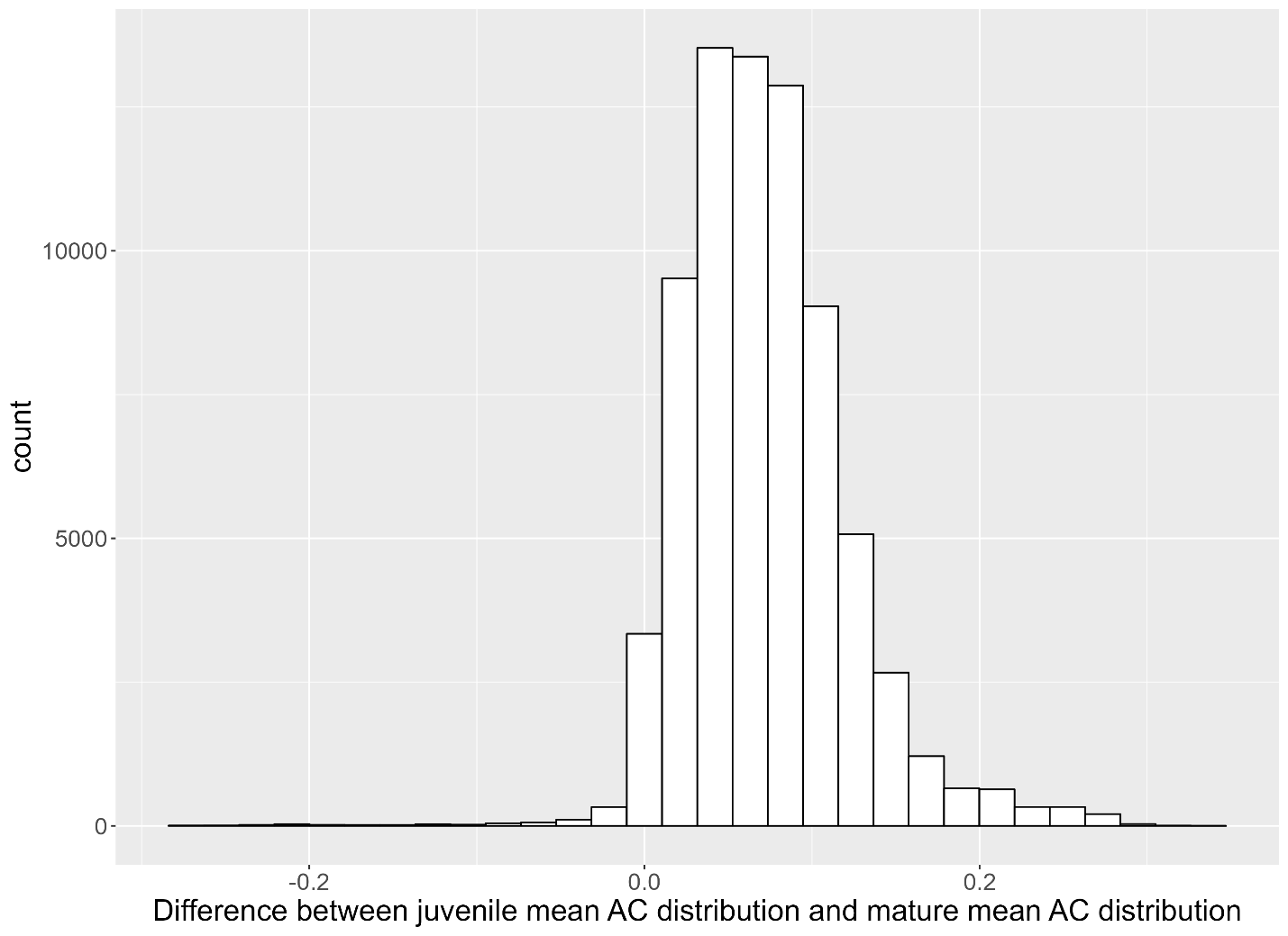
*

*Figure S8. Distribution of the difference in posterior distributions of mean activity centers between mature and juvenile individuals.*

*
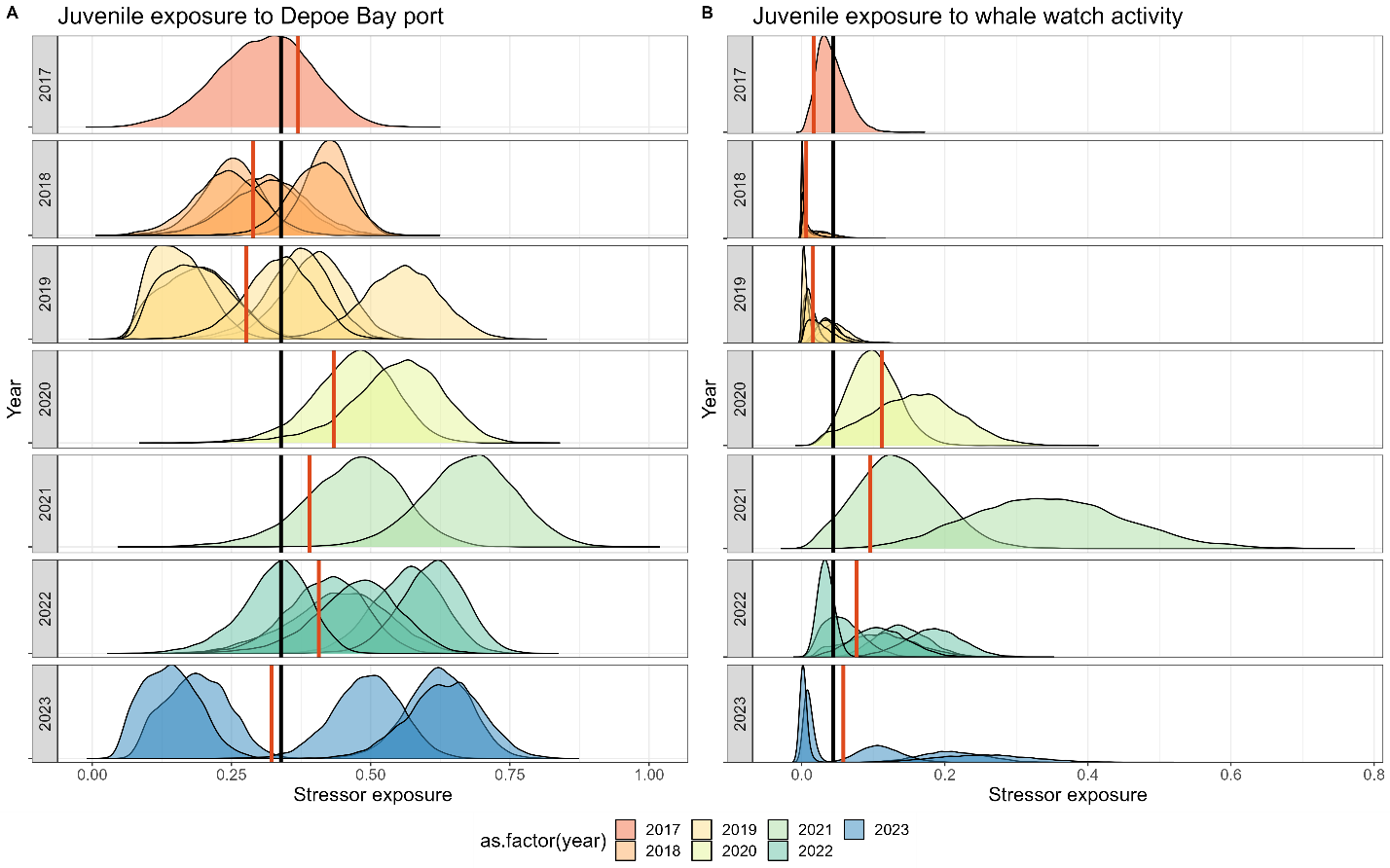
*

*Figure S9. Individual exposure of juvenile gray whales to the port of Depoe Bay (A) and whale watch activity (B). Each line represents an individual’s exposure. The black vertical lines represent the mean of all individuals’ median exposure to each stressor across years and the orange vertical lines represent the annual mean of all individuals’ median exposure to each stressor.*

# References

Barlow, D. R., Strong, C. S., & Torres, L. G. (2024). Three decades of nearshore surveys reveal long-term patterns in gray whale habitat use, distribution, and abundance in the Northern California Current. *Scientific Reports*, *14*(1), 9352.

Derville, S., Buell, T. V., Corbett, K. C., Hayslip, C., & Torres, L. G. (2023). Exposure of whales to entanglement risk in Dungeness crab fishing gear in Oregon, USA, reveals distinctive spatio-temporal and climatic patterns. *Biological Conservation*, *281*, 109989.

Lang, A. R., Calambokidis, J., Scordino, J., Pease, V. L., Klimek, A., Burkanov, V. N., ... & Taylor, B. L. (2014). Assessment of genetic structure among eastern North Pacific gray whales on their feeding grounds. *Marine Mammal Science*, *30*(4), 1473-1493.

Pirotta, E., Bierlich, K. C., New, L., Hildebrand, L., Bird, C. N., Fernandez Ajó, A., & Torres, L. G. (2024). Modeling individual growth reveals decreasing gray whale body length and correlations with ocean climate indices at multiple scales. *Global Change Biology*, *30*(6), e17366.

Rice, D. W., Wolman, A. A. (1971). The life history and ecology of the gray whale (*Eschrichtius robustus*). Special Publication No. 3. American Society of Mammalogists, Stillwater, OK.

Soledade Lemos, L., Burnett, J. D., Chandler, T. E., Sumich, J. L., & Torres, L. G. (2020). Intra‐and inter‐annual variation in gray whale body condition on a foraging ground. *Ecosphere*, *11*(4), e03094.
